# A multi-view latent variable model reveals cellular heterogeneity in complex tissues for paired multimodal single-cell data

**DOI:** 10.1093/bioinformatics/btad005

**Published:** 2023-01-09

**Authors:** Yuwei Wang, Bin Lian, Haohui Zhang, Yuanke Zhong, Jie He, Fashuai Wu, Knut Reinert, Xuequn Shang, Hui Yang, Jialu Hu

**Affiliations:** School of Computer Science, Northwestern Polytechnical University, Shaanxi 710129, China; School of Computer Science, Northwestern Polytechnical University, Shaanxi 710129, China; School of Computer Science, Northwestern Polytechnical University, Shaanxi 710129, China; School of Computer Science, Northwestern Polytechnical University, Shaanxi 710129, China; Department of Biostatistics, School of Public Health, Peking University Health Science Center, Beijing 100191, China; Department of Orthopaedics, Union Hospital, Tongji Medical College, Huazhong University of Science and Technology, Wuhan 430022, China; Institut für Informatik, Freie Universität Berlin, 14195 Berlin, Germany; School of Computer Science, Northwestern Polytechnical University, Shaanxi 710129, China; School of Life Science, Northwestern Polytechnical University, Shaanxi 710072, China; School of Computer Science, Northwestern Polytechnical University, Shaanxi 710129, China

## Abstract

**Motivation:**

Single-cell multimodal assays allow us to simultaneously measure two different molecular features of the same cell, enabling new insights into cellular heterogeneity, cell development and diseases. However, most existing methods suffer from inaccurate dimensionality reduction for the joint-modality data, hindering their discovery of novel or rare cell subpopulations.

**Results:**

Here, we present VIMCCA, a computational framework based on variational-assisted multi-view canonical correlation analysis to integrate paired multimodal single-cell data. Our statistical model uses a common latent variable to interpret the common source of variances in two different data modalities. Our approach jointly learns an inference model and two modality-specific non-linear models by leveraging variational inference and deep learning. We perform VIMCCA and compare it with 10 existing state-of-the-art algorithms on four paired multi-modal datasets sequenced by different protocols. Results demonstrate that VIMCCA facilitates integrating various types of joint-modality data, thus leading to more reliable and accurate downstream analysis. VIMCCA improves our ability to identify novel or rare cell subtypes compared to existing widely used methods. Besides, it can also facilitate inferring cell lineage based on joint-modality profiles.

**Availability and implementation:**

The VIMCCA algorithm has been implemented in our toolkit package *scbean* (≥0.5.0), and its code has been archived at https://github.com/jhu99/scbean under MIT license.

**Supplementary information:**

Supplementary data are available at *Bioinformatics* online.

## 1 Introduction

Thanks to advances in single-cell sequencing technologies, we can quantitatively characterize gene expression (Tang *et al.*, 2009), transposase-accessible chromatin (Buenrostro *et al.*, 2015), methylation (Luo *et al.*, 2018) and other modalities (Nagano *et al.*, 2013) at single-cell resolution. Due to their high-throughput and high-resolution properties, these fast-growing technologies have attracted ever-increasing interest over the past decade. Enormous single-cell datasets have been generated across tissues, organs and species, which include, but are not limited to, HCA (Regev *et al.*, 2017), MCA (Han *et al.*, 2018), single-cell atlas of chromatin accessibility in both human (Zhang *et al.*, 2021) and mouse (Cusanovich *et al.*, 2018). These comprehensive datasets, along with many computational and statistical single-cell analysis tools (Hu *et al.*, 2021a, b), have significantly facilitated the understanding of cellular heterogeneity (Kiselev *et al.*, 2019), cell development (Treutlein *et al.*, 2014), coding and non-coding genetic variants associated with traits (Poirion *et al.*, 2018) and diseases (Chung *et al.*, 2017; Mathys *et al.*, 2019). However, these single-mode assays can potentially lose biological signals due to their modality-specific technical noises. Data from each modality provide a unique view of cellular biology, but each has its strengths and weaknesses. For example, sub-cell types of T cells are difficult to be separated by profiling RNA alone (Mereu *et al.*, 2020). Thus, some recent cutting-edge single-cell technologies [e.g. CITE-seq (Stoeckius *et al.*, 2017), SNARE-seq (Chen *et al.*, 2019), 10× multiome, scNMT-seq (Clark *et al.*, 2018) and sci-CAR (Cao *et al.*, 2018)] have been developed to simultaneously measure two or more data modalities from the same cells, and some can even retain spatial cellular context (Moffitt *et al.*, 2016; Sylwestrak *et al.*, 2016). Although these multimodal single-cell technologies provide invaluable opportunities to interrogate cells from multiple modalities, integrating this new data is still challenging due to the need to infer a common source of variations from the different modalities (Miao *et al.*, 2021). Thus, it is increasingly in demand to develop effective integration methods that can accurately define detailed cellular maps on multi-modality data of diverse tissues and facilitate integrated analyses to uncover cellular heterogeneity, functional regulator and cell state transition in normal and pathological contexts.

Existing integration methods for multi-modal data can be grouped into two categories by the types of input data: methods for unpaired data and methods for paired data. Many methods have been developed for integrating unpaired data across modalities, which mainly focused on transferring cell type annotation of a reference scRNA-seq data onto cells of another modality, including Seurat v3 (Stuart *et al.*, 2019), LIGER (Welch *et al.*, 2019) and its updated version iNMF (Gao *et al.*, 2021), VIPCCA (Hu *et al.*, 2021b) and other methods based on variational inference (Hu *et al.*, 2021a). With the emergence of the joint-modality platform, there is a critical need for a novel computational tool to integrate paired multimodal omics data simultaneously measured in the same cells. To do so, a pioneering tool, Seurat v4 (Hao *et al.*, 2021) was proposed to integrate two modality-specific graphs into one single graph using a strategy of weighted-nearest neighbor (WNN), which calculates cell-specific modality weights for each cell in each modality and determines its relative information content for each cell. A statistical framework MOFA+ (Argelaguet *et al.*, 2020) was developed to infer latent factors and associated feature weight matrices based on variational inference techniques and group-wise automatic relevance determination (ARD), thus enabling simultaneous integration of multiple modalities and sample groups. More recent methods include variants of variational autoencoder (VAE) [e.g. Cobolt (Gong *et al.*, 2021), scVAE (Grønbech *et al.*, 2020), scVI (Lopez *et al.*, 2018), peakVI (Ashuach *et al.*, 2022), scMVAE (Zuo and Chen, 2021), scMM (Minoura *et al.*, 2021) and totalVI (Gayoso *et al.*, 2021)] that learn a non-linear joint embedding through a multimodal encoder model and BABEL (Wu *et al.*, 2021) that use two encoder models to project either RNA or ATAC profiles into a shared latent representation, decoder models to infer observed phenotypes from the latent representation. More details about existing methods are provided in Supplementary Table S1 and Supplementary Text.

However, most existing methods have a specific weakness that prevents them from analyzing and interpreting cellular heterogeneity based on the joint-modality data. Specifically, Seurat v4 infers a single integrated graph to obtain a common definition of cellular state without a statistical model, thus missing a chance of accounting for the source of variations within each modality. Methods with sophisticated parametric models such as MOFA+ are challenging to be scaled up to large-size data. Although current deep-learning methods such as Cobolt and totalVI can be scaled up to large-size data, they have the potential to mask the presence of rare or small subpopulations of cells due to the inaccurate prediction of low-dimensional cell representations. BABEL (Wu *et al.*, 2021) is unable to directly integrate multi-modal data for downstream analysis such as joint-clustering analysis, while it can use joint-modality data to predict one single modality into another modality. The variants of deep generative models such as scMVAE, scMM and totalVI take features of each modality as input to learn joint latent representation. Many successful applications demonstrate their abilities to facilitate integrating single-cell multi-omics data. However, most of the above methods focus on integrating only one type of multi-modal data. For example, Seurat v4 and totalVI were mainly designed to integrate CITE-seq that jointly measures gene expression and a few hundred antibodies for the same cells.

To overcome these challenges, we propose a unified computational framework, VIMCCA, based on variational inference and multi-view subspace learning to understand cell identity and function from paired multi-modal omics. In contrast to existing algorithms, we assume a multi-view latent variable *Z* exists to interpret the source of variations within each modality. Our model projected the single latent factor *Z* into multi-modal observation spaces by modality-specific non-linear functions.

## 2 Materials and methods

### 2.1 A multi-view latent variable model

Suppose, we have a joint-modality data D={X,Y}, where $X\in R^{n\times p}$ represents a gene expression matrix of *n* cells measured on *p* genes, and $Y\in R^{n\times q}$ represents a matrix of the same *n* cells measured on either surface proteins or chromatin-accessible region. Both *X* and *Y* are scaled to unit variance and zero mean by Scanpy (Wolf *et al.*, 2018). We assume that the observed data {*X*, *Y*} can be generated from a latent factor *Z* with two modality-specific non-linear functions. The lower-dimensional matrix *Z* of dimensionality *n* by *d* is supposed to reflect the actual biological states of cells. It could facilitate many downstream analyses such as identifying cell subpopulations and trajectory inference, clustering and visualization. To estimate *Z*, we model the joint-modality data into a non-linear model that transforms a latent variable *z* into the two observed spaces. Mathematically, it can be described by a statistical model as written below:

(1) $$x_{i}=f_{x}(z_{i};\theta_{x})+\epsilon_{x}$$

(2) $$y_{i}=f_{y}(z_{i};\theta_{y})+\epsilon_{y}$$

(3) $$z_{i}∼N(0,I_{d})\;\;\epsilon_{x}∼N(0,\sigma^{2}I_{p})\;\;\epsilon_{y}∼N(0,\sigma^{2}I_{q})$$

where $x_{i}$ and $y_{i}$ are the joint-modality data of the *i*th cell; *f_x_* and *f_y_* are two non-linear regression functions that transform $z_{i}$ from a latent *d*-dimensional space into two observed spaces, respectively; *θ_x_* and *θ_y_* are parameters which can be estimated by fitting the given data; and *ϵ_x_* and *ϵ_y_* are the residual errors that follow multivariate Gaussian distribution. We assume that the prior distribution of the latent variable *z_i_* is a standard multivariate normal distribution $N(0,I_{d})$. Under this assumption, the latent representation vector of the *n* cells all reside in the same lower-dimensional space. The non-linear function $f_{x}(\cdot )$ and $f_{y}(\cdot )$ are constructed by using two generative deep neural network structures (i.e. decoder). The decoders take *z_i_* as input and output reconstructed matrices *x_i_* and *y_i_*, respectively. In the decoder $f_{x}(\cdot )$, *z_i_* is inputted into *d*-dimensional layer, which is then connected to the output *p*-dimensional layer with several intermediate layers in the form of d→32→64→128→p. The structure of $f_{y}(\cdot )$ is the same as $f_{x}(\cdot )$ except for its *q*-dimensional output layer. All intermediate layers are fully connected through a BatchNormalization layer (with centering but no scaling), a Relu activation layer and a Dropout layer (rate = 0.05). The dropout technique can prevent over-fitting and provide a way of efficiently combining exponentially many different neural network architectures. It’s noticed that the strategy of using BatchNormalization and Dropout layers simultaneously in our networks do not lead to worse performance in our data analysis (Supplementary Figs S1 and S2) although they do make some modern networks perform worse in some cases (Li *et al.*, 2019). In the above model, the non-linear function $f_{x}(\cdot )$ and $f_{y}(\cdot )$ generalize the standard probabilistic CCA model (Bach and Jordan, 2005) towards modeling both non-linear and the modality-specific variances, thus allowing us to learn complex features of the joint-modality data.

### 2.2 The variational inference algorithm

As described in Equations (1–3), we assume that the two observed modality data *X* and *Y* are generated by some random process involving a latent variable *z*. However, the true parameters $\theta^{*}=(\theta_{x},\theta_{y},\sigma )$ are unknown to us. Furthermore, the marginal likelihood $p_{\theta }(X,Y)$ is intractable because of the non-linearity of f(·). Thus, to effectively estimate the posterior of the latent variables *z* based on the observed matrice *X* and *Y*, we develop a variational inference algorithm that uses a variational distribution $q_{\phi }(z|X)$ to approximately estimate the posterior $p_{\theta }(X,Y|z)$. Specifically, the marginal data log-likelihood can be written as

(4) $$\text{log}\;p_{\theta }(X,Y)=\sum_{i=1}^{n}\;\text{log}\;p_{\theta }(x_{i},y_{i})$$

with each term expressed as

(5) $$\text{log}\;p_{\theta }(x_{i},y_{i})=\text{log}\;\int p_{\theta }(x_{i},y_{i}|z_{i})p_{\theta }(z_{i})dz_{i}.$$

The above integration can be solved analytically when *f_x_* and *f_y_* are linear functions as in the standard probabilistic canonical correlation analysis [PCCA, (Bach and Jordan, 2005)]. However, when we use deep neural networks to transform $z_{i}$ to $x_{i}$ and $y_{i}$, the likelihood does not have an analytic form. Thus, using the technique of variational distribution $q_{\phi }(z_{i}|x_{i})$ (Kingma and Welling, 2014), we can re-express Equation (5) as

(6) $$\begin{array}{cc} \;\text{log}\;p_{\theta }(x_{i},y_{i}) & =\int (\text{log}\;p_{\theta }(x_{i},y_{i}))q_{\phi }(z_{i}|x_{i})dz_{i}\\ & \geq E_{q_{\phi }(z_{i}|x_{i})}[\text{log}\;\frac{p_{\theta }(x_{i},y_{i},z_{i})}{q_{\phi }(z_{i}|x_{i})}]\\ & \equiv L(\theta ,\phi ;x_{i},y_{i}). \end{array}$$

Now, the maximization of the marginal log-likelihood has been transformed into a problem of maximizing its Evidence Lower Bound (ELBO) $L(\theta ,\phi ;x_{i},y_{i})$. The ELBO can be rewritten as

(7) $$\begin{array}{cc} & L(\theta ,\phi ;x_{i},y_{i})\\ & =\int q_{\phi }(z_{i}|x_{i})\left(\text{log}\;\frac{p(z_{i})}{q_{\phi }(z_{i}|x_{i})}+\text{log}\;p_{\theta }(x_{i}|z_{i})+\text{log}\;p_{\theta }(y_{i}|z_{i})\right)dz_{i}\\ & =-D_{KL}(q_{\phi }(z_{i}|x_{i})||p(z_{i}))+E_{q_{\phi }(z_{i}|x_{i})}[\text{log}\;p_{\theta }(x_{i}|z_{i})+\text{log}\;p_{\theta }(y_{i}|z_{i})]. \end{array}$$

From Equation (7), we can see that ELBO has two components. The first term is the KL divergence between the variational distribution and the prior $p_{\theta }(z_{i})$, which acts as a KL regularizer. The second term is an expected negative reconstruction error of $x_{i}$ and $y_{i}$. To avoid the construction error being too strong or too weak, we adjust the ELBO via a hyperparameter *λ* in the form as

(8) $$\begin{array}{c} L(\theta ,\phi ;x_{i},y_{i})\\ =-D_{KL}(q_{\phi }(z_{i}|x_{i})||p(z_{i}))\\ +\lambda E_{q_{\phi }(z_{i}|x_{i})}[\text{log}\;p_{\theta }(x_{i}|z_{i})+\text{log}\;p_{\theta }(y_{i}|z_{i})]. \end{array}$$

To estimate the lower bound $L(\theta ,\phi ;x_{i},y_{i})$, we use the SGVB estimator (Kingma and Welling, 2013), which applies Monte Carlo estimates to the variational lower bound. More algorithmic details about the computation of the KL term and the construction error in Equation (8) are described in the Supplementary Text.

### 2.3 10× Multiome RNA+ATAC data on human PBMCs

Our first real dataset was obtained by 10× Multiome from human peripheral blood mononuclear cells (PBMCs) of a healthy donor aged 25. It can be freely downloaded from the 10× Genomics single-cell portal (https://support.10xgenomics.com/single-cell-multiome-atac-gex/datasets/1.0.0/pbmc_granulocyte_sorted_10k), which consists of 10 142 cells (after filtration) with common measurements on 36 601 genes and 106 056 open chromatin peaks. First, we used a function pp.filter_genes (with min_cells = 1) in Scanpy (v1.9.1) (Wolf *et al.*, 2018) to filter low-quality genes and peaks. The count matrices of the remaining genes and peaks were processed with log  transformation. A pseudo count of 1 was added for all elements of the count matrices to avoid taking the logarithm of zeros. Then, the log -normalized data were scaled with a function pp.scale in Scanpy with the default parameter setting. To validate the cell type assignment, we created a pseudo-bulk RNA-seq profile by pooling cells of each assigned cell type. We then compared the pseudo-bulk RNA-seq data with the bulk RNA-seq data of human immune cell types in PBMCs (Memory B, Naive B, CD14 Mono, CD16 Mono, CD4 Naive, CD4 TEM, CD8 Naive, CD8 TEM, gdT, MAIT, NK, pDC, Plasma and Treg), which were downloaded from the GEO website [ID: GSE94820 (Villani et al., 2017). For each cell type, we computed the Pearson correlation coefficients of gene expression of a set of 60 marker genes between the pseudo-bulk RNA-seq data and the bulk RNA-seq data.

### 2.4 10× single-cell immune profiling data on human PBMCs

Our second application downloaded a single-cell immune profiling dataset of human PBMCs from the 10× Genomics website (https://support.10xgenomics.com), which measured 33 538 genes and 17 cell surface proteins on 8258 cells simultaneously. We used a function filter_genes (min_cells = 1) in Scanpy for the gene expression count data to filter low-quality genes. After the filtration, the count matrices of the remaining genes and the 17 proteins were further processed with log-normalization and pp.scale.

### 2.5 CITE-seq data on human bone marrow

In the third application, we applied VIMCCA to a CITE-seq dataset downloaded from the GEO repository (ID: GSE128639) (Stuart *et al.*, 2019). These data consist of 30 672 cells with common measurements on 17 009 genes and 25 cell surface proteins (antibodies). For the scRNA-seq data, we used Scanpy to normalize the expression count data following the standard tutorial of Scanpy. To filter the low-quality data, we first used pp.filter_genes to filter genes with the parameter min_cells = 10, which means that genes expressed in <10 cells will be removed. We then performed sequentially count_per_million (CPM), log-transform, and pp.scale on the RNA count matrix. After the above preprocessing, the gene expression count matrix becomes smooth and follows a Gaussian distribution with unit variance and zero means. We also performed the above preprocessing for the ADT count data without filtering any proteins.

### 2.6 Fetal forebrain and adult forebrain dataset of mouse

In this application, we use a mouse’s fetal forebrain and adult forebrain dataset with cell development stage information. The dataset includes 29 386 genes, 25 845 cells and 2 637 315 open chromatin peaks and can be obtained from GEO (GSE130399). For single-model analysis, we first filter the genes according to the condition that the non-zero count in cells is greater than 3. Then, we use the NormalizeData function of Seurat for standardization with method=LogNormalize and scale.factor = 10 000. We use the ScaleData function for the PCA method to scale the data to unit variance and zero mean.

## 3 Results

### 3.1 Methods overview

**Fig. 1.**
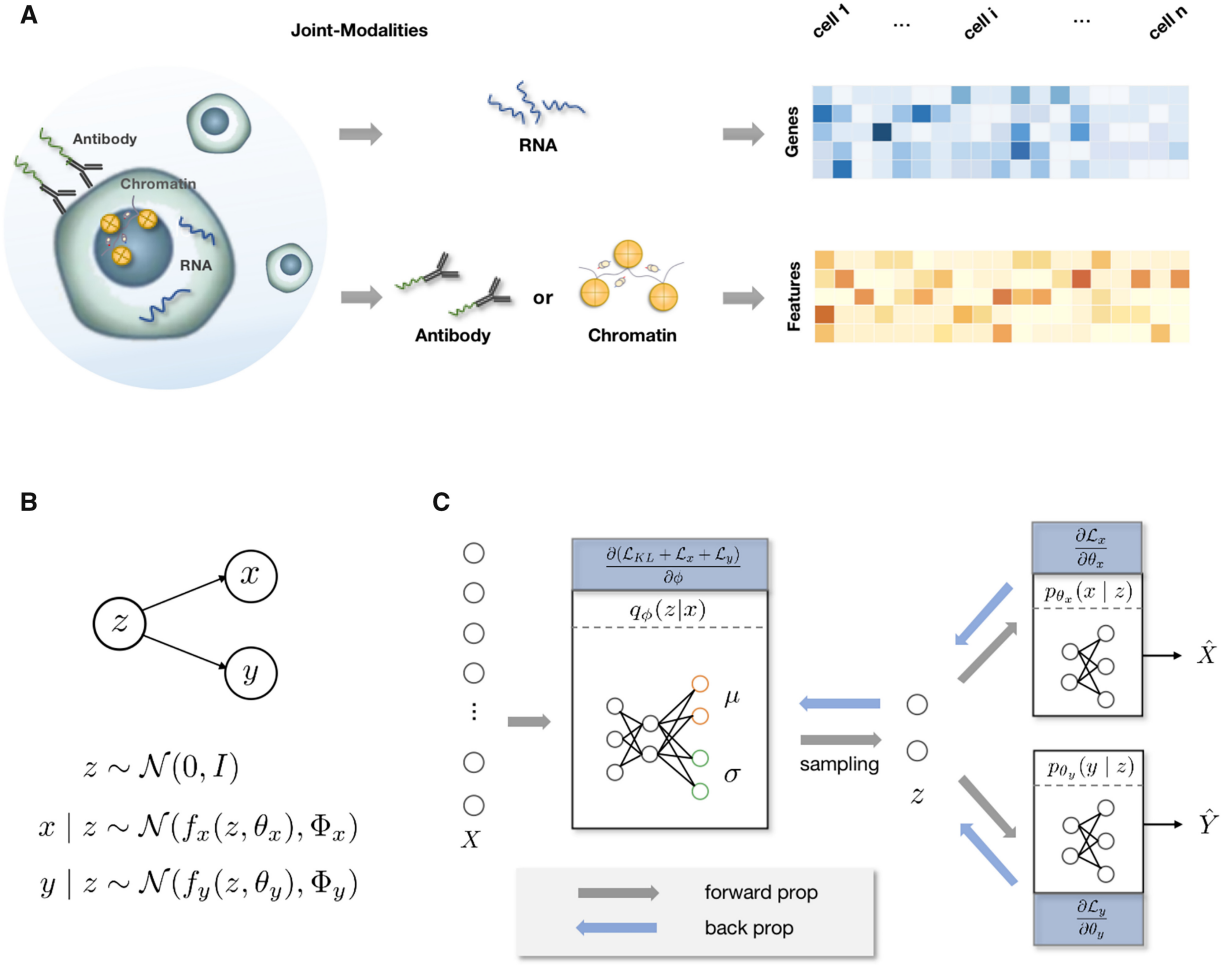
An schematic overview of VIMCCA. (**A**) Input data obtained by single-cell multimodal sequencing technologies are jointly measured on two modalities, such as RNA, antibody or accessibility of chromatin regions. (**B**) A diagram of the multi-view latent variable model of VIMCCA. (**C**) VIMCCA fits a joint-modality data {*X*, *Y*} into an inference model (left) and two non-linearity models (right) using deep neural networks

To broaden our understanding of cell heterogeneity in complex tissues and organs, we developed an efficient computational tool that can analyze paired single-cell multimodal data (Fig. 1A) to define cell types jointly. As shown in Figure 1B, we assume that a standard latent random variable can account for variances of two modality data in the observed space. Then, we incorporate two modality-specific nonlinear functions into the commonly used canonical correlation analysis (CCA) and learn a multi-view latent variable model via variational optimization and multilayer neural network backpropagation for approximate inference (Fig. 1C, details in Section 2). Several features distinguish our approach from existing methods for analyzing single-cell joint-modality data, which include (i) its flexibility in learning complex models, thus allowing us to directly integrate raw peak counts of scATAC-seq and gene expression of scRNA-seq without converting peak counts into gene activity matrix; (ii) its ability to use only transcriptomics data to approximately infer the posterior distribution of the latent variable; (iii) its ability to integrate not only single-cell transcriptomics data and ATAC-seq data but also single-cell transcriptomics data and antibodies. We develop a variational approximation algorithm for inference of the posterior of a latent variable **z** and employ minibatch-based stochastic gradient descent during the training phase, thus allowing the model to be highly computationally scalable. Since our method is designed based on multi-view learning and variational inference, we refer to our method as variational inference-assisted multi-view CCA (VIMCCA). VIMCCA is freely available at https://github.com/jhu99/scbean (v0.5.0).

### 3.2 Integration of a 10× multiome dataset by VIMCCA reveals three new sub-cell types in human PBMCs

To examine the effectiveness of VIMCCA in integrating multimodal data, we first apply it to a dataset of 10 412 paired human PBMC profiles generated by the 10× Genomics Multiome ATAC+RNA kit. All cells are simultaneously measured on 36 601 genes and 106 056 open chromatin peaks. Our goal is to obtain an optimal estimation of the latent variable **z** that can represent the hidden cellular states and sub-classes.

**Fig. 2.**
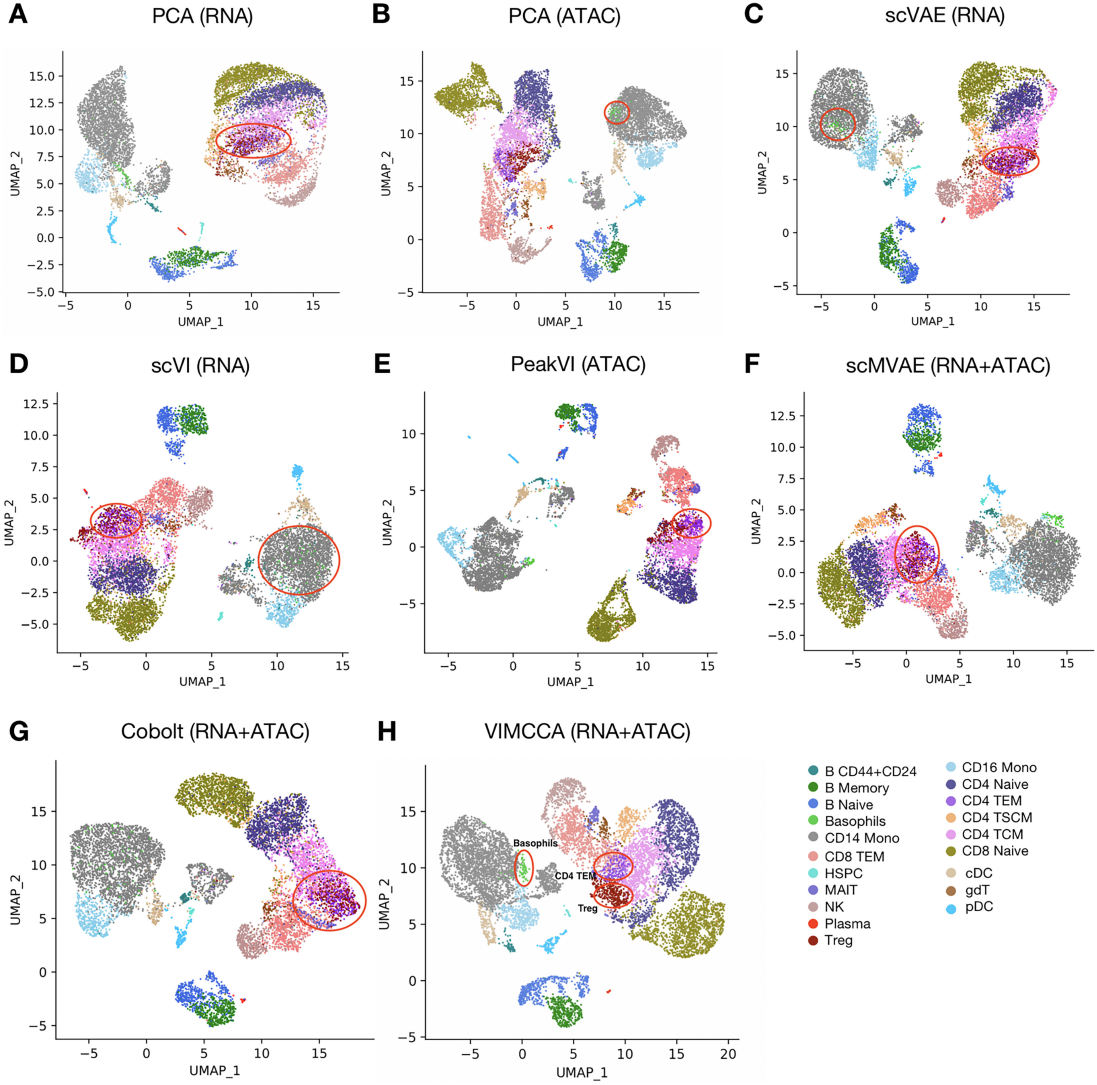
Performance comparison in the lower-dimensional space of 10× multiome RNA+ATAC data. (**A–E**) UMAP visualization of cell representations obtained from either the gene expression data or the ATAC-seq data by PCA, scVAE, scVI and PeakVI. (**F–H**) UMAP visualization of cell representations obtained from both of the two modalities by scMVAE, Cobolt and VIMCCA

To evaluate the performance of VIMCCA and other existing state-of-the-art methods, PCA, scVAE and scVI are performed on only the gene expression data, PCA and PeakVI on only the ATAC data, scMVAE, Cobolt and VIMCCA on both of the two modalities to jointly learn the cell states. UMAP (Becht *et al*., 2019) is used to visualize the cell representation in the reduced dimensional space. From Figure 2A–H, we can see that all compared methods are unable to separate CD4${}^{+}$ T Effector Memory (CD4 TEM) cells and regulatory T cells (Treg), except for PCA (ATAC) and VIMCCA (RNA+ATAC), and Basophil cells cannot be distinguished from CD14${}^{+}$ Monocyte (CD14 Mono) cells by PCA (ATAC), scVAE (RNA), scVI (RNA) and Cobolt (RNA+ATAC). After the integration of the two complementary modalities by performing VIMCCA, all cells can be visually recognized in the UMAP visualization (Fig. 2H).

**Fig. 3.**
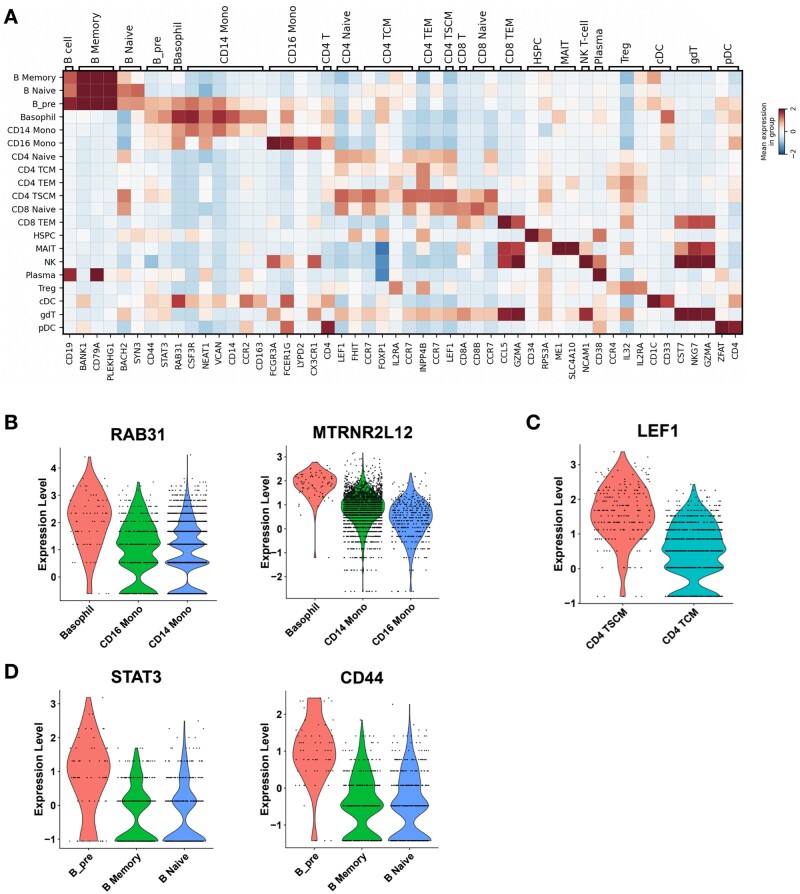
Gene expression patterns of marker genes in the 10× multiome data of human PBMCs. (**A**) A heatmap of cell-type specific marker genes of each cell type. (**B–D**) Expression patterns of marker genes in new sub-populations, such as Basophil, CD4 TSCM and pre-B cells

To evaluate how well our method can define cell states based on both modalities, clustering is performed on the integrated data of VIMCCA using the Leiden algorithm, and 42 clusters are identified (Supplementary Fig. S3). Cell type labels are examined manually based on the prior knowledge of marker features (Thul *et al.*, 2017; Uhlen *et al.*, 2019) (details in Supplementary Table S2). As shown in Figure 3A, all marker genes show significant quantitative changes in expression levels between one representative group and the resting cell type groups (more details in Supplementary Fig. S4). It demonstrates that cluster-based cell-type labels identified by VIMCCA are consistent with the gene expression patterns of the previously reported marker genes. More importantly, three rare sub-cell types, including Basophil cells, CD4 TSCM cells and pre-B cells, have been identified by VIMCCA. In Seurat’s annotation, most Basophil cells are assigned to CD14 Mono and CD16 Mono, CD4 TSCM cells to CD4 TCM, pre-B cells to B Memory and B Naive. However, as shown in Figure 3B–D, the expression of known marker genes including RAB31, MTRNR2L2, LEF1, STAT3 and CD44 (Ding *et al.*, 2016; Horst *et al.*, 1990; Thul *et al.*, 2017; Uhlen *et al.*, 2019) in these newly discovered cell types are significantly higher than the expression in the original cell types.

**Fig. 4.**
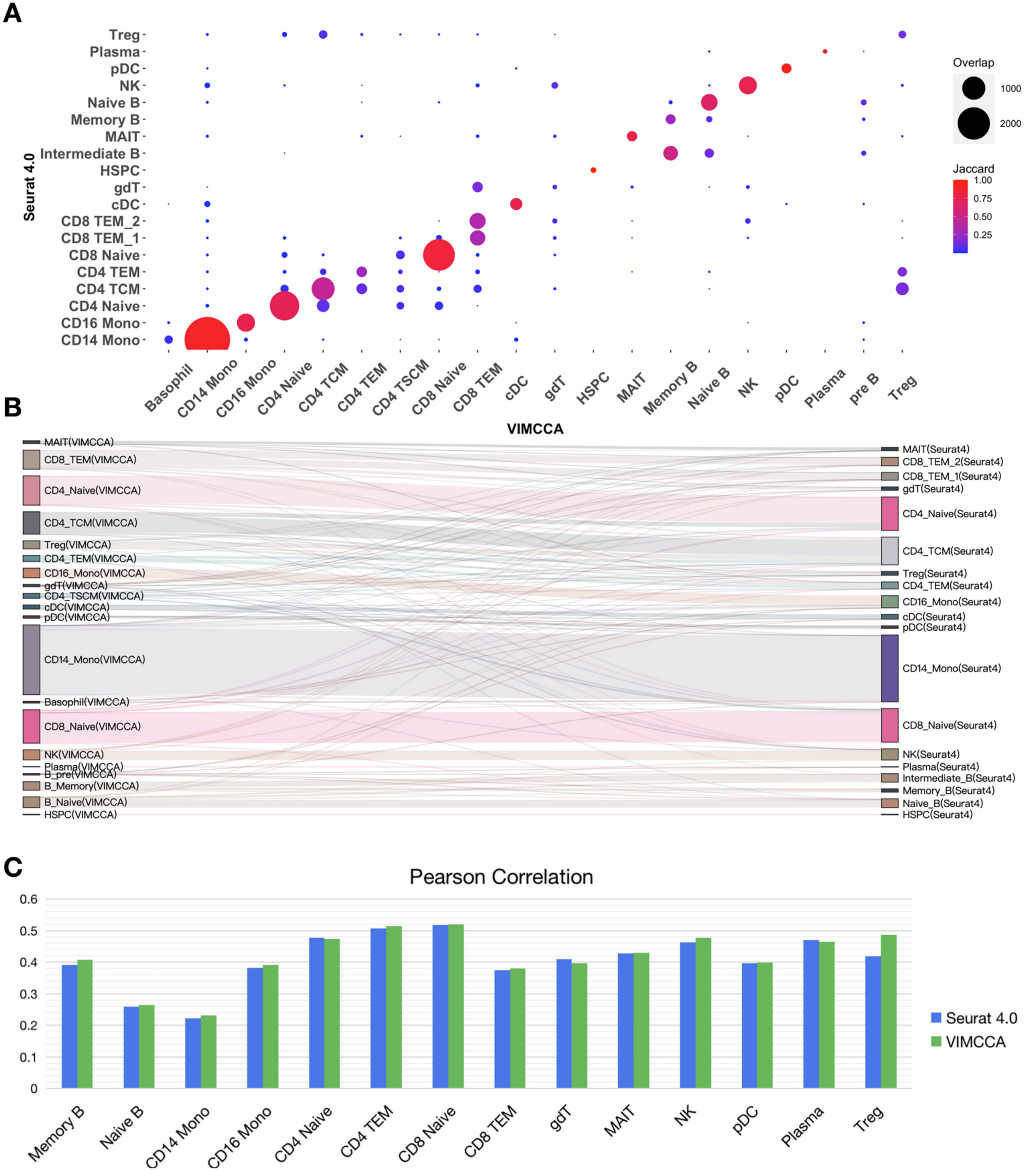
Comparison of cell-type labels annotated by Seurat v4 and VIMCCA. (**A**) Comparison of each pair of cellular subpopulations identified by Seurat v4 and VIMCCA. (**B**) A Sankey diagram depicts a flow from the cell-type labels annotated by VIMCCA to that by Seurat. (**C**) Pearson correlation between the pseudo-bulk RNA-seq profile and the bulk RNA-seq profile

We next perform a comprehensive comparison for pairs of cell types defined by VIMCCA and Seurat v4 in terms of overlapped cells and Jaccard similarity. As shown in Figure 4A, the two sets of cell labels, to a great extent, are consistent with each other, although with some differences in several small cellular groups, such as Treg, gdT and Memory B. A more clear visualization in a Sankey diagram (Fig. 4B) shows the flow direction from labels in VIMCCA to their cell type labels in Seurat v4. To quantitatively measure the accuracy of cell type labels obtained by VIMCCA and Seurat v4, we collect bulk RNA-seq data (Villani *et al.*, 2017) for 14 overlapped cell types, including Memory B, Naive B, CD14 Mono, CD16 Mono, CD4 Naive, CD4 TEM, CD8 Naive, CD8 TEM, gdT, MAIT, NK, pDC, Plasma and Treg, and compute the Pearson correlation between gene expression in each scRNA-seq cellular population and its corresponding cellular group in bulk RNA-seq data. As shown in Figure 4C, cell type labels of VIMCCA are more accurate than that of Seurat v4 in almost all the 14 cellular populations in terms of Pearson correlation. Overall, VIMCCA can capture mutually exclusive characteristics of multi-modal data, thus leading to a more reliable and accurate cell type annotation for human PBMCs.

### 3.3 VIMCCA enables the integration of joint-modality data with both gene expression and cell surface proteins

**Fig. 5.**
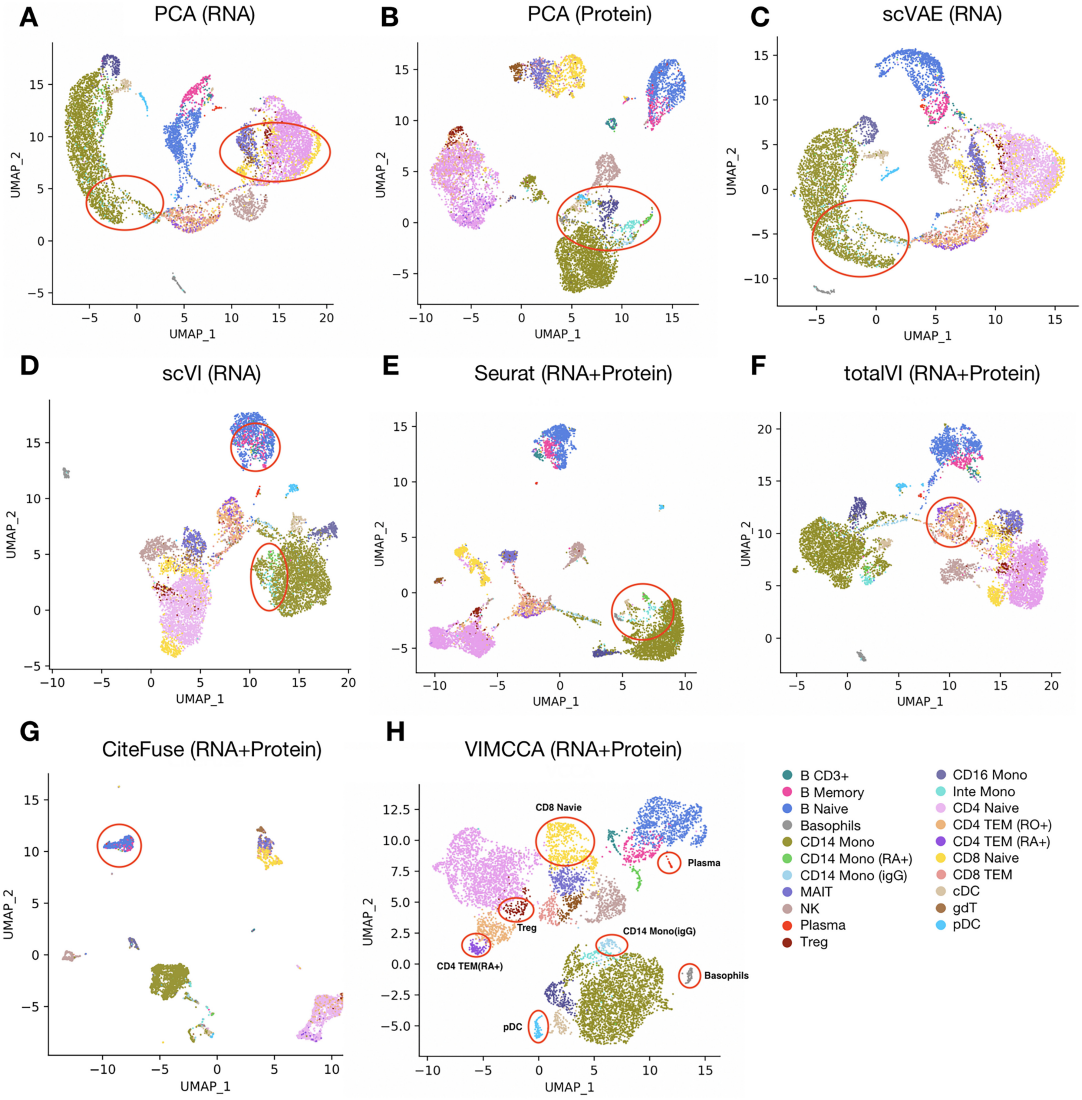
Performance comparison in the lower-dimensional space of 10× single-cell immune profiling of human PBMCs. (**A–D**) UMAP visualization of cell representations obtained from either the gene expression data or the cell surface protein data by PCA, scVAE and scVI. (**E–H**) UMAP visualization of cell representations obtained from both of the two modalities by Seurat, totalVI, CiteFuse and VIMCCA

**Fig. 6.**
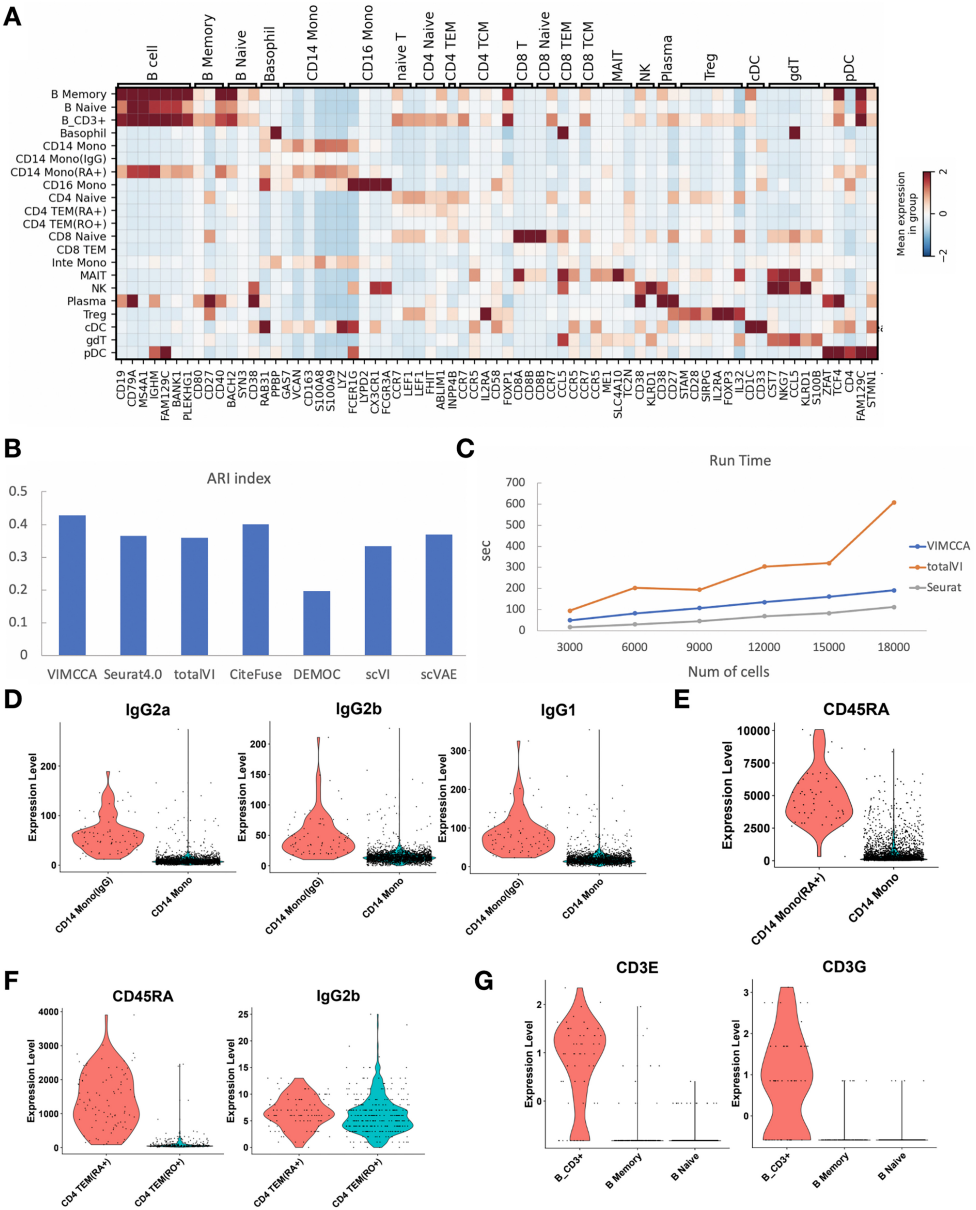
Expression patterns of marker genes and antibodies of human PBMCs. (**A**) A heatmap of the expression of cell type-specific marker genes. (**B**) Comparison of ARI of clustering results obtained by VIMCCA, Seurat v4, totalVI, CiteFuse and DEMOC. (**C**) Comparison of running time of VIMCCA, Seurat v4 and totalVI on six datasets with 3000, 6000, 9000, 12 000, 15 000 and 18 000 cells, respectively. (**D**) The expression levels of IgG2a and IgG2b in IgG${}^{+}$ CD 14 Monocyte cells and CD14 Monocyte cells. (**E**) The expression levels of CD45RA in CD45RA${}^{+}$ CD14 Monocyte cells and CD14 Monocyte cells. (**F**) The expression levels of CD45RA and IgG2b in CD45RA${}^{+}$ CD4 TEM cells and RO${}^{+}$ CD4 TEM cells. (**G**) The expression levels of CD3E and CD3G in CD3${}^{+}$ B cells, Naïve B cells and memory B cells

Our second application integrates joint-modality data obtained by 10× single-cell immune profiling. To verify the effectiveness of our methods in integrating the two complementary modalities, we consider the task of integrating two data modalities of 8258 peripheral blood mononuclear cells (PBMCs) measured on 33 538 genes and 17 cell surface proteins (i.e. antibodies). For a fair comparison, PCA, scVAE and scVI are performed on the gene expression data, and PCA on the protein data, VIMCCA along with Seurat v4, TotalVI and CiteFuse (Kim *et al.*, 2020a) on the two complementary modalities to jointly learn the cell representation in the reduced dimensional space. Following the previous application, we use UMAP to visualize the cell representations obtained by each method (Fig. 5A–H). Cluster-based cell-type labels are manually assigned to cells based on a list of previously reported marker genes and differential gene expression analysis (details in Fig. 6A, D–G, Supplementary Table S3). By doing so, a total of 21 cellular populations have been identified. The UMAP visualization demonstrates that igG${}^{+}$ CD14 Monocyte (CD14 Mono igG) cells and CD8 Naïve cells are difficult to be identified by using only gene expression data (Fig. 5A, C and D), and Basophil cells, Plasma cells and pDC cells cannot be identified by using only antibodies (Fig. 5B). The results also show that Seurat, totalVI and CiteFuse fail to distinguish intermediate Monocyte (Inte Mono) and CD4 TEM RA${}^{+}$ from CD14 Mono and CD4 TEM RO+ cells, respectively (Fig. 5E–G). In the results of totalVI, CD8 Naïve cells are divided into three distinct groups, and Treg cells are mixed with CD4 Naïve cells (Fig. 5E). In contrast, all these cell types can be successfully identified after the integration of VIMCCA (Fig. 5H).

We next evaluate algorithm performance with an adjusted rand index (ARI), which assesses how well cells can be correctly clustered into the 21 different cellular populations. As shown in Figure 6B, VIMCCA outperforms other compared algorithms with the highest ARI value (0.42), which is followed by CiteFuse (0.39), Seurat (0.36), totalVI (0.35) and DEMOC (0.18). Additionally, we test the computational efficiency and scalability of the compared methods on six datasets with 3000, 6000, 9000, 12 000, 15 000 and 18 000 cells, respectively. It shows that VIMCCA, totalVI and Seurat can complete all the integration tasks within about 10 min (Fig. 6C); CiteFuse and DEMOC need more than 24 h to integrate 15 000 cells (Supplementary Table S5). The fast speed of VIMCCA and totalVI majorly benefit from using the neural network structures, and a strategy of mini-batch gradient descent in the deep learning model. More importantly, four new sub-cell types [i.e. IgG${}^{+}$ CD14 Mono (Calverley *et al.*, 2004), CD45RA${}^{+}$ CD14 Mono (Rothe *et al.*, 1996), CD45RO${}^{+}$ CD4 TEM (Richards *et al.*, 1997), CD3${}^{+}$ B (Nagel *et al.*, 2014)] have been discovered by VIMCCA, which can be further verified by the expression patterns of several known marker genes including IgG2a, IgG2b, IgG1, CD45RA, CD3E and CD3G (Fig. 6D–G). Overall, VIMCCA enables efficient integration of joint-modality data generated by 10× single-cell immune profiling, thus leading to more reliable downstream clustering analysis.

### 3.4 Accurate integration of CITE-seq data by VIMCCA improves our ability to resolve cell heterogeneity of human bone marrow cells

**Fig. 7.**
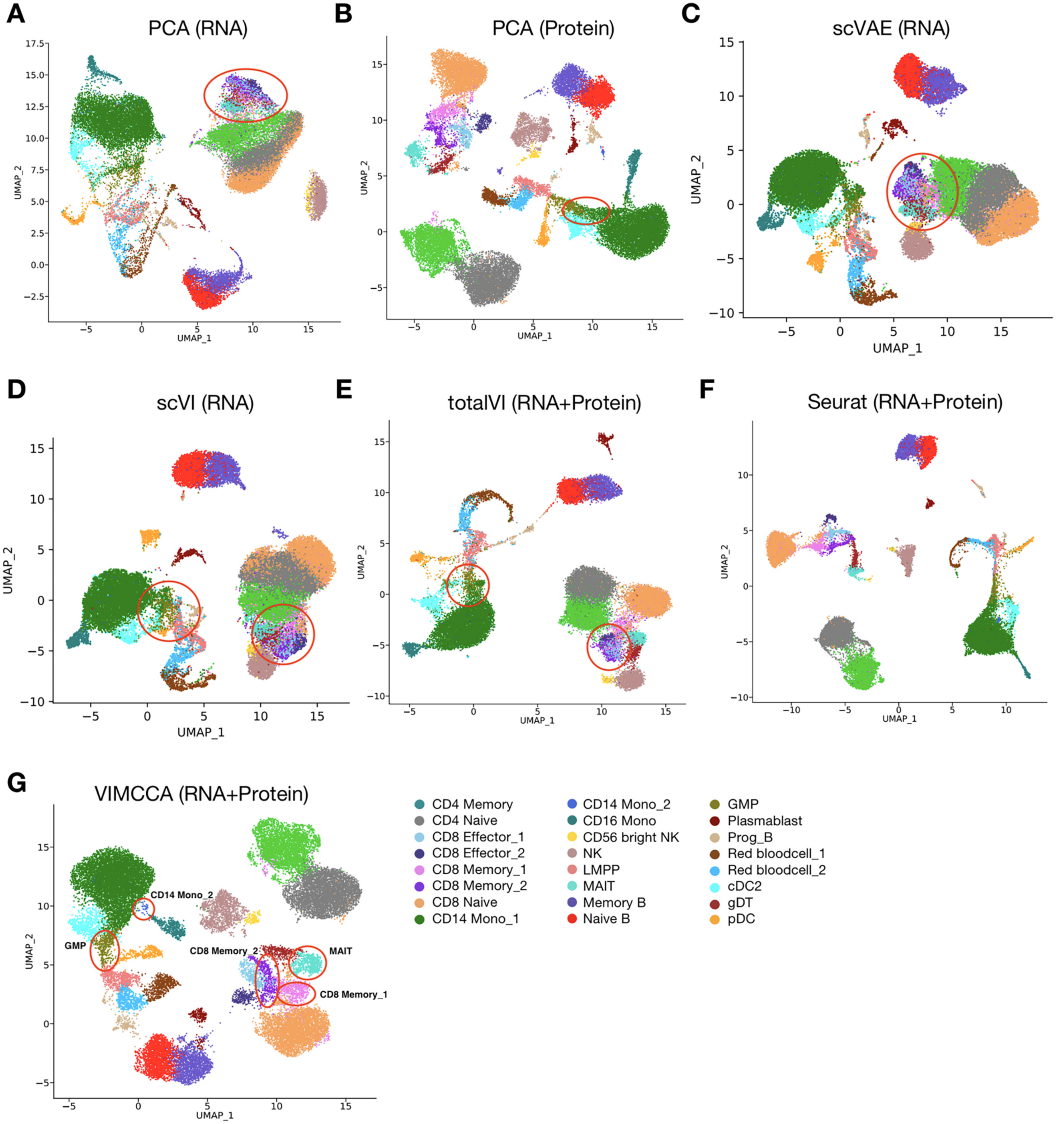
Integrated analyses of CITE-seq data of human bone marrow cells. (**A–D**) UMAP visualization of cell representations obtained from either the gene expression data or the protein surface data by PCA, scVAE and scVI. (**F, G**) UMAP visualization of cell representations obtained from both of the two modalities by totalVI, Seurat and VIMCCA

**Fig. 8.**
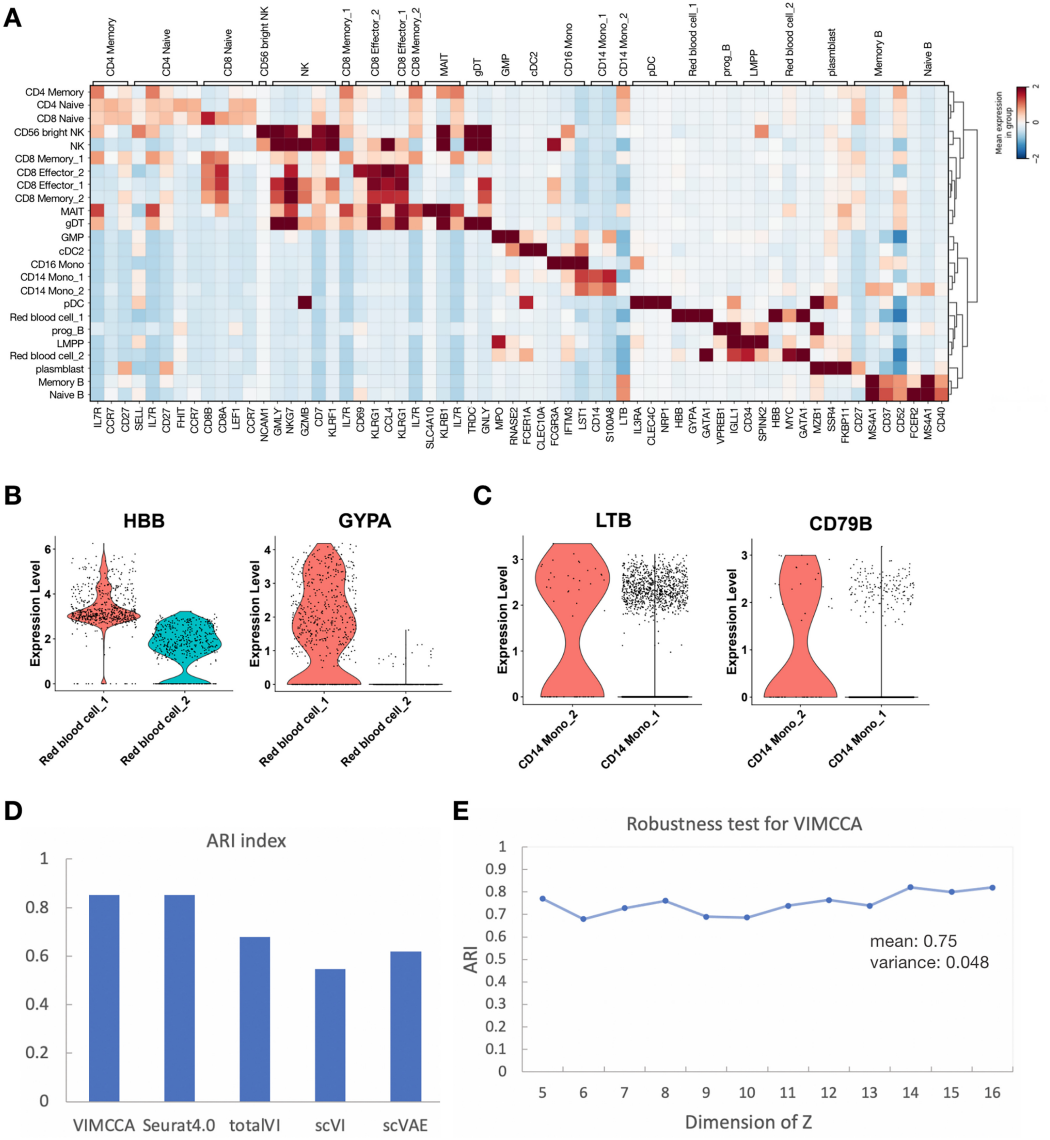
Expression patterns of marker genes and performance evaluation based on ARI for human bone marrow cells. (**A**) A heatmap of expression patterns of marker genes of each cell type. (**B**) The expression levels of HBB and GYPA in red blood 1 and red blood 2. (**C**) The LTB and CD79B expression levels in CD14 Monocyte 1 and CD14 Monocyte 2. (**D**) ARI index of clustering result of VIMCCA, Seurat v4 and totalVI on human bone marrow cells. (**E**) Robustness analysis for the hyperparameter of VIMCCA

In our third application, we examine the task of integrating data generated from a CITE-seq experiment (Stoeckius *et al.*, 2017; Stuart *et al.*, 2019), in which 17 009 genes and a panel of 25 cell surface proteins were simultaneously measured on 30 672 human bone marrow cells. For performance comparison, we applied PCA (RNA), PCA (Protein), scVAE (RNA), scVI (RNA), totalVI (RNA+Protein), Seurat (RNA+Protein) and VIMCCA (RNA+Protein) to this dataset, and cell-type labels are manually assigned to cells based on clustering analysis and differential expression analysis. UMAP is used to visualize the cell representation obtained by the compared methods (Fig. 7A–G), and a total of 24 cell types are identified. To do this, more than 60 cell-type-specific marker genes are collected and curated from literature (Supplementary Table S4), and their expression patterns are used for cluster-based cell type annotations (Fig. 8A). Results show that CD8 Memory 1 cannot be distinguished from CD8 Memory 2 and Mucosa-associated invariant T (MAIT) cells by PCA (RNA), scVAE (RNA), scVI (RNA) and totalVI (Fig. 7A, C, D and E); most existing methods cannot distinguish granulocyte-monocyte progenitors (GMP) cells and CD14 Monocyte 2 (CD14 Mono_2) cells from the major CD14 Mono 1 cells (Fig. 7A–F). By taking advantage of both the information from RNA and antibodies with a multi-view model, VIMCCA accurately identified most of the 24 cell types, including a rare sub-cell type CD14 Mono 2 (Fig. 7G).

**Fig. 9.**
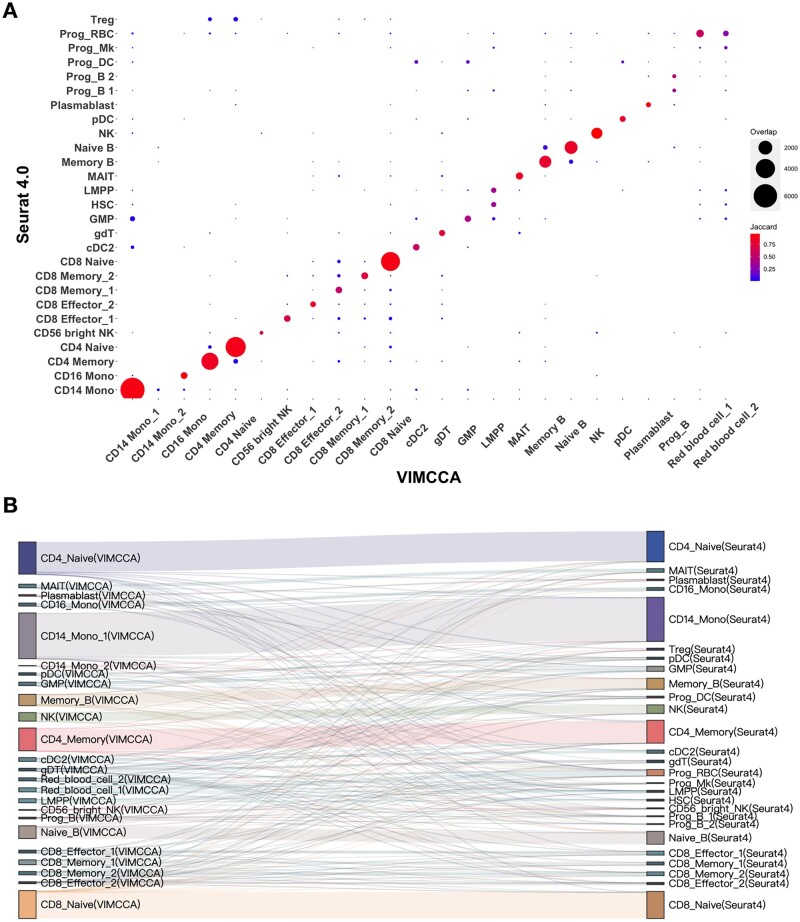
Expression patterns of marker genes and performance evaluation based on ARI for human bone marrow cells. (**A**) Comparison of each pair of cellular subpopulations identified by Seurat v4 and VIMCCA. (**B**) A Sankey diagram depicts a flow from the cell-type labels annotated by VIMCCA to that by Seurat

We next compare the cell-type annotation of VIMCCA with that of Seurat v4. As shown in Figure 9A and B, most of the cell type labels predicted by VIMCCA are consistent with that predicted by Seurat, except for red blood 1 and red blood 2, and CD14 Monocyte 2. Most red blood cells are recognized as regulatory T cells and progenitor red blood cells, and CD14 Monocyte 2 is a rare subpopulation newly discovered by VIMCCA. All these findings are further verified by the expression patterns of previously reported marker genes HBB, GYPA, LTB and CD79B (Fig. 8B and C). HBB and GYPA are marker genes of red blood cells (Young *et al.*, 2018). At the same time, they show different expression patterns in these two subtypes (Fig. 8B), indicating that these two subtypes are two different subpopulations of red blood cells. The different expression patterns of LTB and CD79B in CD14 Mono 1 and CD14 Mono 2 show that these cells are two subpopulations of CD14 Mono cells (Fig. 8C). We further evaluate the performance of clustering in terms of ARI. Results show that the clustering result of VIMCCA is comparable to that of Seurat v4 and is superior to that of totalVI, scVAE and scVI (Fig. 8D). In addition, we measure the robustness of the choice of the number of latent dimensions (Fig. 8E). It demonstrates that our clustering results are robust with respect to the dimensionality of *Z*. Overall, VIMCCA facilitates the integration of CITE-seq data across gene expression data and cell surface proteins, thus improving our ability to identify rare sub-cell types.

### 3.5 VIMCCA integration enables accurate trajectory inference for the development of fetal forebrain

Trajectory inference algorithms used in the single-cell analysis are to determine the dynamic changes of thousands of cells over time by analyzing the single-cell multi-omics data. Its a major challenge to develop an integration algorithm that can embed two data modalities of cells in latent space while preserving cell trajectory structures. To examine the ability to support downstream trajectory inference, we test VIMCCA, DCA, scVI, scVAE, PCA and Diffusion map on a time-series single-cell dataset, which was collected from 25 845 samples (cells) of E12.5, E16.5 and adult mouse fetal forebrain (Zhu *et al.*, 2019) on 29 386 genes and 2 637 315 open chromatin peaks. Note that VIMCCA leverages both two modalities to reduce the dimensionality. After the dimension reduction, we perform slingshot (v1.8.0) (Street *et al.*, 2018) on cell embeddings in the lower dimensional space to infer pseudotime and cellular lineage within the developing forebrain.

**Fig. 10.**
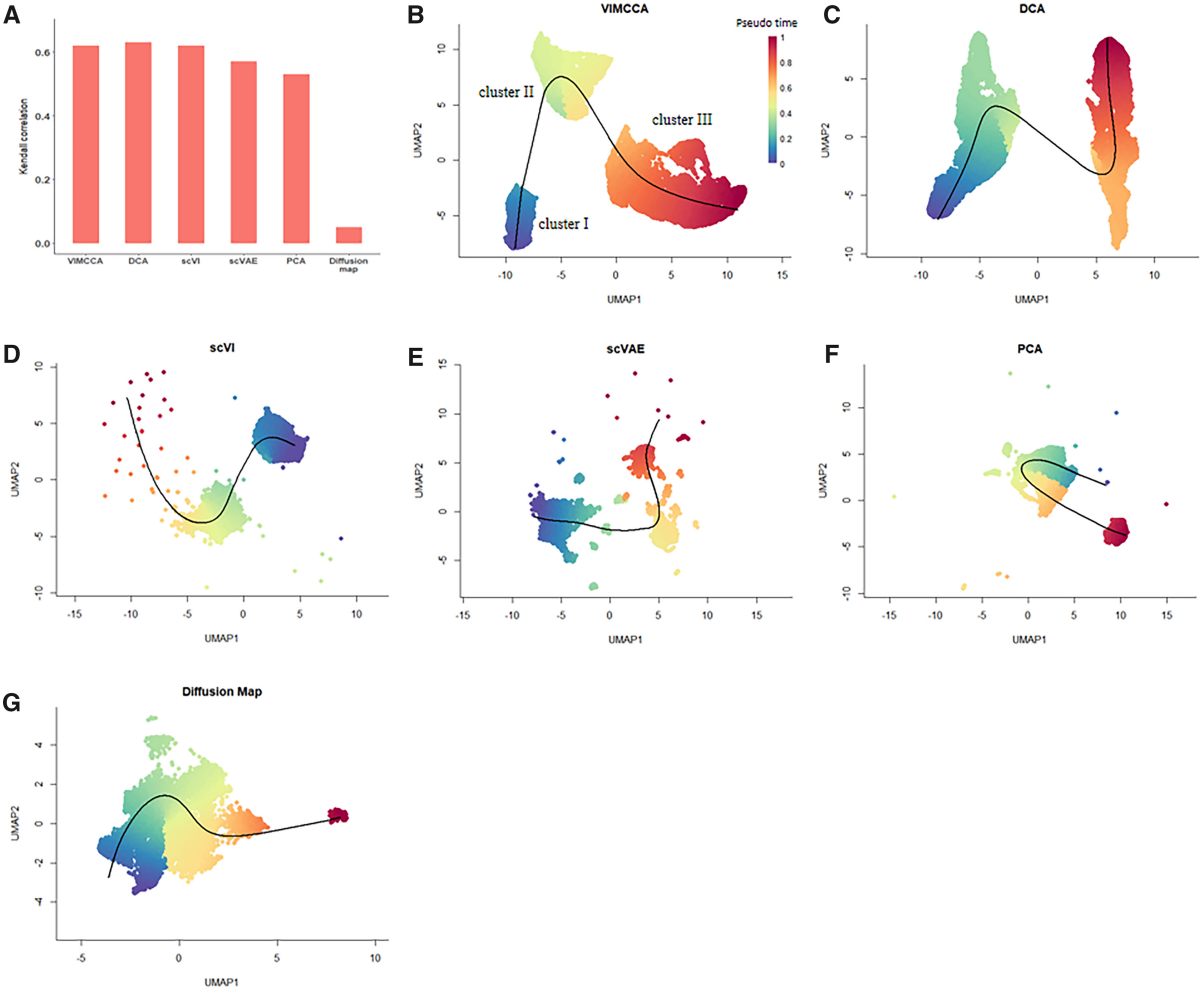
Trajectory inference analysis for the development of mouse fetal forebrain based on the reduced dimensionality obtained by various methods. (**A**) The Kendall correlation coefficient between the real sampling time of cells and the pseudo-time inferred by the eight methods. (**B–I**) Comparison of the trajectory inferred by VIMCCA, DCA, scVI, scVAE, PCA, t-SNE, UMAP and diffusion map

For a fair performance comparison, we evaluate the integration quality of the compared algorithms in terms of the Kendall correlation (*τ*) between the inferred pseudotime and the real-time cell states. As shown in Figure 10A, quantification supports the superior performance of DCA, VIMCCA, and scVI in terms of the correlation. Their correlation scores are 0.63, 0.62 and 0.62, respectively, which are followed by scVAE (0.55), PCA (0.50) and Diffusion map (0.04). UMAP visualization in Figure 10B–G demonstrates that VIMCCA is the only algorithm that can identify three distinct clusters. Cells in each cluster represent a cell state during the mouse forebrain development, although cells in Clusters I and II are mixed by cells from E12.5 and E16.5 mouse forebrain (Supplementary Fig. S6). To understand the biological role of each identified cluster, we further provide differential expression analysis and gene set enrichment analysis for each cluster. As a result, 130, 75 and 77 cluster-specific genes are identified for Clusters I–III, respectively. Then we perform enrichment analysis on the three gene sets in biological process (Supplementary Fig. S7) and cellular component, respectively (Supplementary Fig. S8). Results show that cells in Clusters I–III play major roles in synapse organization, neuron migration and dendrite development. Previous studies also demonstrate that newborn neurons migrate to specific positions in the brain and extend axons and dendrites before engaging in synapse formation during the mouse forebrain development (Südhof, 2021, 2018). Overall, we can conclude that VIMCCA facilitates accurate trajectory inference by integrating time-series multi-modal single-cell data.

## 4 Discussion

Our method VIMCCA uses a multiview non-linear CCA model to jointly learn the common cell embeddings from paired multimodal single-cell data. Seurat v4 performs a dimensional reduction of each modality separately and merges two modality-specific distance matrices into one single distance matrix via weighted-nearest neighbor (WNN). Through our manually annotated cell labels and careful and comprehensive evaluation, we demonstrate that VIMCCA facilitates the integration of various types of multimodal data, thus leading to accurate and robust downstream analysis, such as the identification of new cellular subpopulations and trajectory inference. However, although these cell labels are verified by the expression patterns of previously reported markers, we cannot guarantee that these labels are 100% correct, so they may slightly affect the results. Further, we take the single-cell transcriptomics data as the encoder’s input to search for a Gaussian approximation of the posterior distribution of the latent variable in all three real data applications. Considering another type of modality data in the input layer may also affect the integration quality.

## 5 Conclusions

Advanced single-cell multimodal sequencing technologies provide new opportunities to understand cell heterogeneity from multiple modalities. However, most existing methods may encounter limitations while applied to various multimodal datasets, which consequently limit the quality of downstream analyses. It poses novel computational challenges in integrating two or more distinct modalities from single-cell sequencing.

To provide potential solutions to improve the integration quality, we propose a unified computational framework, VIMCCA, based on a combination of variational inference and a multi-view latent model, which can learn the cell representations in the lower-dimensional space from paired multi-modal omics. To verify the effectiveness and stability of VIMCCA, we apply VIMCCA and several state-of-the-art methods to four real paired multi-modal datasets generated by four different technologies or protocols. Results suggest that VIMCCA can improve our ability to integrate multimodal data by taking advantage of the complementary information, thus leading to more accurate identification of cell types and rare sub-cell types. Compared with existing methods, VIMCCA’s contributions are 4-fold: (i) the unified computational framework based on multi-view non-linear CCA models enables VIMCCA to account for the common source of variations for single-cell multi-modal data; (ii) the non-linearity modeling enables VIMCCA to capture complex data structures of various multimodal datasets, including 10× multiome, 10× single-cell immune profiling, CITE-seq data and Paired-seq; (iii) by taking advantage of variational inference, VIMCCA implements computationally efficient algorithms, thus leading to more reliable, scalable and robust downstream analysis; (iv) the multi-view non-linear CCA model enables VIMCCA to directly learn cell representations from the peak counts of ATAC-seq and the transcriptomics data without translating the peak counts into the activity matrix.

We anticipate that VIMCCA will be widely used for the conjoint analysis of various multimodal data. For example, VIMCCA can be easily extended to integrate multimodal data measured simultaneously on three or more modalities. Specifically, it only requires more modality-specific neural network layers as additional decoders in the computational framework. It is expected to enable more accurate and robust cell type definition and downstream analysis than these sequenced from two modalities. For example, it has the potential to be used for discovering gene regulation mechanism from omics data (Zhang *et al.*, 2022a, b).

Like some existing single-cell analysis methods (Argelaguet *et al.*, 2020; Hu *et al.*, 2021a; Xin *et al.*, 2020), VIMCCA mainly focuses on modeling gene expression data through a Gaussian noise model. Modeling raw transcriptome count data using the Gaussian model is computationally more tractable than using over-dispersed or zero-inflated Poisson models [e.g. negative binomial, Poisson mixture models, zero-inflated negative binomial, etc. (Sun *et al.*, 2017; Sun *et al.*, 2018)].

Due to the relatively low sequencing depth of single-cell sequencing, accounting for mean and variance relationships by directly modeling raw count data often provides additional benefits (Kim *et al.*, 2020b). Therefore, adopting a zero-inflated Poisson distribution to model the data of gene transcriptome modalities may further improve the quality of the cell embedding features learned by VIMCCA.

## Supplementary Material

btad005_Supplementary_Data

## Data Availability

No new data were generated or analysed in support of this research.

## References

[btad005-B1] Argelaguet R. et al (2020) MOFA+: a statistical framework for comprehensive integration of multi-modal single-cell data. Genome Biol., 21, 17. 32393329 10.1186/s13059-020-02015-1 PMC7212577

[btad005-B2] Ashuach T. et al (2022) PeakVI: a deep generative model for single-cell chromatin accessibility analysis. Cell Rep. Methods, 2, 100182. 35475224 10.1016/j.crmeth.2022.100182 PMC9017241

[btad005-B3] Bach F.R. , Jordan M.I. (2005) A probabilistic interpretation of canonical correlation analysis. In: *Technical Report 688, Department of Statistics*. University of California, Berkeley.

[btad005-B29] Becht,E. et al. (2019) Dimensionality reduction for visualizing single-cell data using UMAP. *Nat. Biotechnol*., 37, 38–44. 10.1038/nbt.4314 30531897

[btad005-B4] Buenrostro J.D. et al (2015) Single-cell chromatin accessibility reveals principles of regulatory variation. Nature, 523, 486–490. 26083756 10.1038/nature14590 PMC4685948

[btad005-B5] Calverley D.C. et al (2004) Association between monocyte Fcγ subclass expression and acute coronary syndrome. Immunity Ageing, 1, 4. 15679933 10.1186/1742-4933-1-4 PMC544957

[btad005-B6] Cao J. et al (2018) Joint profiling of chromatin accessibility and gene expression in thousands of single cells. Science, 361, 1380–1385. 30166440 10.1126/science.aau0730 PMC6571013

[btad005-B7] Chen S. et al (2019) High-throughput sequencing of the transcriptome and chromatin accessibility in the same cell. Nat. Biotechnol., 37, 1452–1457. 31611697 10.1038/s41587-019-0290-0 PMC6893138

[btad005-B8] Chung W. et al (2017) Single-cell RNA-seq enables comprehensive tumour and immune cell profiling in primary breast cancer. Nat. Commun., 8, 15081–15012. 10.1038/ncomms15081 PMC5424158 28474673

[btad005-B9] Clark S.J. et al (2018) scNMT-seq enables joint profiling of chromatin accessibility DNA methylation and transcription in single cells. Nat. Commun., 9, 1–9. 29472610 10.1038/s41467-018-03149-4 PMC5823944

[btad005-B10] Cusanovich D.A. et al (2018) A single-cell atlas of in vivo mammalian chromatin accessibility. Cell, 174, 1309–1324.e18. 30078704 10.1016/j.cell.2018.06.052 PMC6158300

[btad005-B11] Ding C. et al (2016) STAT3 signaling in B cells is critical for germinal center maintenance and contributes to the pathogenesis of murine models of lupus. J. Immunol., 196, 4477–4486. 27183592 10.4049/jimmunol.1502043 PMC4875824

[btad005-B12] Gao C. et al (2021) Iterative single-cell multi-omic integration using online learning. Nat. Biotechnol., 1–8. 33875866 10.1038/s41587-021-00867-x PMC8355612

[btad005-B13] Gayoso A. et al (2021) Joint probabilistic modeling of single-cell multi-omic data with totalVI. Nat. Methods, 18, 272–282. 33589839 10.1038/s41592-020-01050-x PMC7954949

[btad005-B14] Gong B. et al (2021) Cobolt: integrative analysis of multimodal single-cell sequencing data. Genome Biol., 22, 1–21. 34963480 10.1186/s13059-021-02556-z PMC8715620

[btad005-B15] Grønbech C.H. et al (2020) scVAE: variational auto-encoders for single-cell gene expression data. Bioinformatics, 36, 4415–4422. 32415966 10.1093/bioinformatics/btaa293

[btad005-B16] Han X. et al (2018) Mapping the mouse cell atlas by Microwell-seq. Cell, 172, 1091–1107.e17. 29474909 10.1016/j.cell.2018.02.001

[btad005-B17] Hao Y. et al (2021) Integrated analysis of multimodal single-cell data. Cell. 10.1016/j.cell.2021.04.048 PMC8238499 34062119

[btad005-B18] Horst E. et al (1990) Expression of a human homing receptor (CD44) in lymphoid malignancies and related stages of lymphoid development. Leukemia, 4, 383–389. 2201831

[btad005-B19] Hu J. et al (2021a) A versatile and scalable single-cell data integration algorithm based on domain-adversarial and variational approximation. Brief. Bioinform., 23, bbab400. 10.1093/bib/bbab400 34585247

[btad005-B20] Hu J. et al (2021b) Effective and scalable single-cell data alignment with non-linear canonical correlation analysis. Nucleic Acids Res., 50, e21. 10.1093/nar/gkab1147 PMC8887421 34871454

[btad005-B21] Kim H.J. et al (2020a) CiteFuse enables multi-modal analysis of CITE-seq data. Bioinformatics, 36, 4137–4143. 32353146 10.1093/bioinformatics/btaa282

[btad005-B22] Kim T. et al (2020b) Demystifying “drop-outs” in single-cell UMI data. Genome Biol., 21. 10.1186/s13059-020-02096-y PMC7412673 32762710

[btad005-B23] Kingma D.P. , Welling M. (2014) Auto-encoding variational Bayes. *Statistics*, 1050(3), 1.

[btad005-B24] Kiselev V.Y. et al (2019) Challenges in unsupervised clustering of single-cell RNA-seq data. Nat. Rev. Genet., 20, 273–282. 30617341 10.1038/s41576-018-0088-9

[btad005-B25] Li X. et al (2019) Understanding the disharmony between dropout and batch normalization by variance shift. In: *Proceedings of the IEEE/CVF Conference on Computer Vision and Pattern Recognition*, pp. 2682–2690.

[btad005-B26] Lopez R. et al (2018) Deep generative modeling for single-cell transcriptomics. Nat. Methods, 15, 1053–1058. 30504886 10.1038/s41592-018-0229-2 PMC6289068

[btad005-B27] Luo C. et al (2018) Robust single-cell DNA methylome profiling with snmC-seq2. Nat. Commun., 9, 3824. 30237449 10.1038/s41467-018-06355-2 PMC6147798

[btad005-B28] Mathys H. et al (2019) Single-cell transcriptomic analysis of Alzheimer’s disease. Nature, 570, 332–337. 31042697 10.1038/s41586-019-1195-2 PMC6865822

[btad005-B30] Mereu E. et al (2020) Benchmarking single-cell RNA-sequencing protocols for cell atlas projects. Nat. Biotechnol., 38, 747–755. 32518403 10.1038/s41587-020-0469-4

[btad005-B31] Miao Z. et al (2021) Multi-omics integration in the age of million single-cell data. Nat. Rev. Nephrol., 17, 710–724. 34417589 10.1038/s41581-021-00463-x PMC9191639

[btad005-B32] Minoura K. et al (2021) A mixture-of-experts deep generative model for integrated analysis of single-cell multiomics data. Cell Rep. Methods, 1, 100071. 35474667 10.1016/j.crmeth.2021.100071 PMC9017195

[btad005-B33] Moffitt J.R. et al (2016) High-performance multiplexed fluorescence in situ hybridization in culture and tissue with matrix imprinting and clearing. Proc. Natl. Acad. Sci. USA, 113, 14456–14461. 27911841 10.1073/pnas.1617699113 PMC5167177

[btad005-B34] Nagano T. et al (2013) Single-cell Hi-C reveals cell-to-cell variability in chromosome structure. Nature, 502, 59–64. 24067610 10.1038/nature12593 PMC3869051

[btad005-B35] Nagel A. et al (2014) CD3-positive B cells: a storage-dependent phenomenon. PLoS One, 9, 1–10. 10.1371/journal.pone.0110138 PMC4199681 25329048

[btad005-B36] Poirion O. et al (2018) Using single nucleotide variations in single-cell RNA-seq to identify subpopulations and genotype-phenotype linkage. Nat. Commun., 9, 1–13. 30459309 10.1038/s41467-018-07170-5 PMC6244222

[btad005-B37] Regev A. et al (2017) Science forum: the human cell atlas. Elife, 6, e27041. 29206104 10.7554/eLife.27041 PMC5762154

[btad005-B38] Richards D. et al (1997) Immune memory in CD4+ CD45RA+ T cells. Immunology, 91, 331–339. 9301520 10.1046/j.1365-2567.1997.00274.x PMC1364000

[btad005-B39] Rothe G. et al (1996) Peripheral blood mononuclear phagocyte subpopulations as cellular markers in hypercholesterolemia. Arterioscler. Thromb. Vasc. Biol., 16, 1437–1447. 8977447 10.1161/01.atv.16.12.1437

[btad005-B40] Stoeckius M. et al (2017) Simultaneous epitope and transcriptome measurement in single cells. Nat. Methods, 14, 865–868. 28759029 10.1038/nmeth.4380 PMC5669064

[btad005-B41] Street K. et al (2018) Slingshot: cell lineage and pseudotime inference for single-cell transcriptomics. BMC Genomics, 19, 1–16. 29914354 10.1186/s12864-018-4772-0 PMC6007078

[btad005-B42] Stuart T. et al (2019) Comprehensive integration of single-cell data. Cell, 177, 1888–1902.e21. 31178118 10.1016/j.cell.2019.05.031 PMC6687398

[btad005-B43] Südhof T.C. (2018) Towards an understanding of synapse formation. Neuron, 100, 276–293. 30359597 10.1016/j.neuron.2018.09.040 PMC6226307

[btad005-B44] Südhof T.C. (2021) The cell biology of synapse formation. J. Cell Biol., 220, e202103052. 34086051 10.1083/jcb.202103052 PMC8186004

[btad005-B45] Sun S. et al (2018) Heritability estimation and differential analysis of count data with generalized linear mixed models in genomic sequencing studies. Bioinformatics, 35, 487–496. 10.1093/bioinformatics/bty644 PMC6361238 30020412

[btad005-B46] Sun S. et al (2017) Differential expression analysis for RNAseq using Poisson mixed models. Nucleic Acids Res., 45, e106. 28369632 10.1093/nar/gkx204 PMC5499851

[btad005-B47] Sylwestrak E.L. et al (2016) Multiplexed intact-tissue transcriptional analysis at cellular resolution. Cell, 164, 792–804. 26871636 10.1016/j.cell.2016.01.038 PMC4775740

[btad005-B48] Tang F. et al (2009) mRNA-Seq whole-transcriptome analysis of a single cell. Nat. Methods, 6, 377–382. 19349980 10.1038/nmeth.1315

[btad005-B49] Thul P. J. et al (2017) A subcellular map of the human proteome. Science, 356, eaal3321. 28495876 10.1126/science.aal3321

[btad005-B50] Treutlein B. et al (2014) Reconstructing lineage hierarchies of the distal lung epithelium using single-cell RNA-seq. Nature, 509, 371–375. 24739965 10.1038/nature13173 PMC4145853

[btad005-B51] Uhlen M. et al (2019) A genome-wide transcriptomic analysis of protein-coding genes in human blood cells. Science, 366, eaax9198. 31857451 10.1126/science.aax9198

[btad005-B52] Villani A.C. et al (2017) Single-cell RNA-seq reveals new types of human blood dendritic cells, monocytes, and progenitors. Science, 356, eaah4573. 28428369 10.1126/science.aah4573 PMC5775029

[btad005-B54] Welch J.D. et al (2019) Single-cell multi-omic integration compares and contrasts features of brain cell identity. Cell, 177, 1873–1887.e17. 31178122 10.1016/j.cell.2019.05.006 PMC6716797

[btad005-B55] Wolf F.A. et al (2018) SCANPY: large-scale single-cell gene expression data analysis. Genome Biol., 19, 1–5. 29409532 10.1186/s13059-017-1382-0 PMC5802054

[btad005-B56] Wu K.E. et al (2021) Babel enables cross-modality translation between multiomic profiles at single-cell resolution. Proc. Natl. Acad. Sci. USA, 118, e2023070118. 10.1073/pnas.2023070118 PMC8054007 33827925

[btad005-B57] Xin H. et al (2020) GMM-Demux: sample demultiplexing, multiplet detection, experiment planning, and novel cell-type verification in single cell sequencing. Genome Biol., 21, 1–35. 10.1186/s13059-020-02084-2 PMC7393741 32731885

[btad005-B58] Young M.D. et al (2018) Single-cell transcriptomes from human kidneys reveal the cellular identity of renal tumors. Science, 361, 594–599. 30093597 10.1126/science.aat1699 PMC6104812

[btad005-B59] Zhang K. et al (2021) A single-cell atlas of chromatin accessibility in the human genome. Cell, 184(24), 5985–6001. 10.1016/j.cell.2021.10.024 PMC8664161 34774128

[btad005-B60] Zhang P. et al (2022a) CLNN-loop: a deep learning model to predict CTCF-mediated chromatin loops in the different cell lines and CTCF-binding sites (CBS) pair types. Bioinformatics, 38, 4497–4504. 35997565 10.1093/bioinformatics/btac575

[btad005-B61] Zhang P. et al (2022b) iPro-WAEL: a comprehensive and robust framework for identifying promoters in multiple species. Nucleic Acids Res., 50, 10278–10289. 36161334 10.1093/nar/gkac824 PMC9561371

[btad005-B62] Zhu C. et al (2019) An ultra high-throughput method for single-cell joint analysis of open chromatin and transcriptome. Nat. Struct. Mol. Biol., 26, 1063–1070. 31695190 10.1038/s41594-019-0323-x PMC7231560

[btad005-B63] Zuo C. , Chen L. (2021) Deep-joint-learning analysis model of single cell transcriptome and open chromatin accessibility data. Brief. Bioinform., 22, bbaa287. 33200787 10.1093/bib/bbaa287 PMC8293818

