# Supplementary material for: A multi-view latent variable model reveals cellular heterogeneity in complex tissues for paired multimodal single-cell data
Source: Bioinformatics. 2023 Jan 9;39(1):btad005. doi: 10.1093/bioinformatics/btad005 (PMC9857983; doi:10.1093/bioinformatics/btad005)
Supplement: btad005_Supplementary_Data [file btad005_supplementary_data.pdf]

A multi-view latent variable model reveals cellular heterogeneity in complex tissues for paired multimodal single-cell data

Yuwei Wang[1,†], Bin Lian[1,†], Haohui Zhang[1], Yuanke Zhong[1], Jie He[2], Fashuai Wu[3], Knut Reinert[4], Xuequn Shang[1], Hui Yang[5], and Jialu Hu[1,*]

[1]*School of Computer Science, Northwestern Polytechnical University, 1 Dong Xiang Rd., 710129, Shaanxi, China. Tel: 029-88431519.*
[2]*Department of Biostatistics, School of Public Health, Peking University Health Science Center, 38 Xueyuan Rd., 100191, Beijing, China. Tel: 010-62744196.*
[3]*Department of Orthopaedics, Union Hospital, Tongji Medical College, Huazhong University of Science and Technology, 430022, Wuhan. Tel: 027-83665555.*
[4]*Institut für Informatik, Freie Universität Berlin, Takustr. 9, 14195 Berlin, Germany. Tel: 49-30-83875222.*
[5]*School of Life Science, Northwestern Polytechnical University, 127 West Youyi Rd., 710072, Shaanxi, China. Tel: 029-88460332.*

[*]Correspondence: jhu@nwpu.edu.cn,[†]Co-first authors

Supplementary Text

The KL term

We assume that the posterior of the latent variable $p_\theta(\mathbf{z}_i|\mathbf{x}_i, \mathbf{y}_i)$ follows a multivariate Gaussian distribution with diagonal covariance. It allows us to write the variational distribution as

$$\log q_\phi(\mathbf{z}_i|\mathbf{x}_i) = \log \mathcal{N}(\mathbf{z}_i; \mu_i, \Sigma_i) \tag{1}$$

where $\mu_i = \{\mu_{i1}, \cdots, \mu_{id}\}, \Sigma_i = diag\{\sigma_{i1}^2, \sigma_{i2}^2, \cdots, \sigma_{id}^2\}$. Since the prior $p(\mathbf{z}_i) = \mathcal{N}(\mathbf{z}_i; \mathbf{0}, \mathbf{I}_d)$, we can compute analytically for the KL term in the equation 8 (in the main document) as

$$D_{KL}(q_\phi(z_i|x_i)||p(z_i)) = -\frac{1}{2}\sum_{j=1}^{d}(1 + \log\sigma_{ij}^2 - \sigma_{ij}^2 - \mu_{ij}^2). \tag{2}$$

To estimate the parameters μ_i and Σ_i, we also employ deep neural network layers (i.e. encoder), which take $\mathbf{x}_i$ as inputs, and output two d-vector μ_i and $(\sigma_{i1}^2, \sigma_{i2}^2, \cdots, \sigma_{id}^2)$ through several intermediate neural network layers. The structure of the encoder is in the form of $p \to 128 \to 64 \to 32 \to 2d$.

The expected negative reconstruction error

The second term in equation 8 (in the main document) is an expected log-likelihood under the approximate posterior distribution, also known as an expected negative reconstruction error of X and Y. The hyperparameter λ is used to adjust the pressure of reconstruction error. This term doesn't have a closed-form solution because of the non-linear function $f(\cdot)$. Thus, it requires estimation by sampling. Specifically, for each $\mathbf{z}_i, i = 1, ..., n$, we sample L times from $q_\phi(\mathbf{z}_i|\mathbf{x}_i)$, yielding

$$\begin{aligned}
&\mathcal{L}(\theta, \phi; \mathbf{x}_i, \mathbf{y}_i) \approx \tilde{\mathcal{L}}(\mathbf{x}_i, \mathbf{y}_i; \theta, \phi) \\
&= -D_{KL}(q_\phi(\mathbf{z}_i|\mathbf{x}_i)||p(\mathbf{z}_i)) + \frac{\lambda}{L}\sum_{l=1}^{l=L}(\log p_\theta(\mathbf{x}_i|\mathbf{z}_i^l) + \log p_\theta(\mathbf{y}_i|\mathbf{z}_i^l))
\end{aligned} \tag{3}$$

where $\mathbf{z}_i^l \sim \mathcal{N}(\mu_i, \Sigma_i)$. However, directly sampling random variable $\mathbf{z}_i^l$ from $q_\phi(\mathbf{z}_i|\mathbf{x}_i)$ would result in in-differential bottleneck layer in the deep neural networks. To come up with this problem, we reparameterize the random variable $\mathbf{z}_i \sim q_\phi(\mathbf{z}_i|\mathbf{x}_i)$ using a differentiable transformation of an auxiliary noise variable ϵ as

$$z_i^l = \mu_i + \sigma_i \circ \epsilon^l \tag{4}$$

where $\epsilon^l \sim \mathcal{N}(\mathbf{0}, I_d)$. The symbol $\circ$ represents the element-wise multiplication of two vectors. Since the residual error ϵ_x and ϵ_y are in the standard multivariates Gaussian distribution as defined in the equation **??**-**??**, we can find that

$$\mathcal{L}(\theta, \phi; \mathbf{x}_i, \mathbf{y}_i) \approx \frac{1}{2}\sum_{j=1}^{d}(1 + \log\sigma_{ij}^2 - \sigma_{ij}^2 - \mu_{ij}^2) - \lambda \cdot p(\log(2\pi) + \log\sigma^2)$$
$$- \frac{\lambda}{\sigma^2 L}\sum_{l=1}^{L}(||\mathbf{x}_i - f_x(\mathbf{z}_i;\theta_x)||^2 + ||\mathbf{y}_i - f_y(\mathbf{z}_i;\theta_y)||^2) \quad (5)$$

Thus, we solve the maximization of the log-likelihood probability by jointly learning an approximate posterior distribution $q_\phi(\mathbf{z}_i|\mathbf{x}_i)$ and a generative multi-view latent model $p_\theta(\mathbf{x}_i, \mathbf{y}_i|\mathbf{z}_i)$.

Other methods used for performance comparison

We compare VIMCCA with several existing methods for multimodal joint analysis such as Seurat v4 [1], totalVI [2] and several single-modal analysis methods such as scVI [3], scVAE [4]. Seurat v4 is a weighted-nearest neighbor (WNN) method to integrate multiple modality-specific graphs into one graph and obtain a joint definition of cellular state. WNN uses an unsupervised strategy to learn cell-specific modality weights for each cell. The weights reflect the information content for each modality and determine its relative importance in downstream analyses. We performed the method strictly according to the tutorial provided by Seurat v4 (https://satijalab.org/seurat/articles/weighted_nearest_n as well as its cell type annotation. Another widely used tool, totalVI, is based on a probabilistic model that uses a variational inference technique to learn a conditional probability distribution based on two modalities. We run totalVI through the API provided by scvi-tools [5] (v0.14.5). ScMVAE is a multi-modal integration method based a combination of MVAE and GMM models. CiteFuse is a streamlined package consisting of a suite of tools for doublet detection, modality integration, clustering, differential RNA and protein expression analysis, antibody-derived tag evaluation, ligand-receptor interaction analysis, and interactive web-based visualization of CITE-seq data. DEMOC is a novel deep-embedded multi-omics clustering method that integrates transcriptomic and proteomic data to identify cellular populations. Cobolt (v1.0.1) is a flexible tool for analyzing multi-modality sequencing data. Single-cell variational inference (scVI) is a ready-to-use scalable framework to learn a probabilistic representation for single-cell transcriptomic data. ScVAE (v2.1.4) is a method based on variational auto-encoders for

the analysis of single-cell RNA sequencing data. It directly uses raw count data as input and can robustly estimate the expected gene expression levels and a latent representation for each cell. PeakVI is a deep generative model for analyzing single-cell chromatin accessibility data. ScVI and peakVI are provided by scvi-tool (v0.18). DCA (v0.3.4) is a robust and fast autoencoder-based denoising method for integrating multiple scRNA-seq data. The diffusion map is a spectral method for non-linear dimension reduction and has recently been adapted for the visualization of single-cell expression data. We use the package of SnapATAC to run diffusion map.

Supplementary Tables

Table S1: Summary of a list of existing tools for joint analyses of multi-modal single-cell data.

Tools	Strategies	Data types	Comments
BABEL [6]		scRNA-seq,	unable to generate joint vector
Cobolt [7]	VAE	scRNA-seq,	
MOFA+ [8]	Factor analysis	scRNA-seq, scATAC-seq	time-consuming
scMM [9]	VAE	scRNA-seq	unable to generate joint vector
Seurat 4.0 [1]	weighted MNN	scRNA-seq, scATAC-seq, Protein	high memory
TotalVI [2]	VAE	scRNA-seq, Protein	time-consuming
VIMCCA	VAE	scRNA-seq, scATAC-seq, Protein	

Table S2: Hyperparameters of the compared methods.

Method	Hyperparameters
scVAE[4]	-m:GMVAE, -l:16, -H:100, -w:200, -e:500
scVI[3]	n_hidden:128, n_latent:10, n_layer:1, gene_likelihood:zinb
PeakVI[10]	n_hidden:128, n_latent:13, latent_distribution:normal
scMVAE[9]	share_hidden:128, Type:ZINB, penality:GMM
Cobolt[7]	lr:0.005, n_latent:10, num_epochs:20
Seurat 4.0[1]	k.nn:20, knn.range:200, prune.SNN:1/15, sd.scale:1
totalVI[2]	n_latent:20, gene_likelihood:nb, latent_distribution:normal
CiteFuse[11]	K_knn:20, K_knn_Aff:30, sigma:0.45, t:10

Table S3: Marker genes used for each cell type in the RNA+ATAC data of human PBMCs.

Cell type	Marker gene
B Memory	CD19+, CD79A+, BANK1+
B Naive	CD19+, CD79A+, BANK1+, CD44+, SYN3+
B pre	CD19+, CD79A+, CD44+
Basophil	RAB31+
CD14 Mono	CD14+, CSF3R+, VCAN+
CD16 Mono	FCGR3A+, FCGR1G+
CD4 Naive	CD4+, LEF1+
CD4 TCM	CD4+, CCR7+, IL2RA-, FOXP1+
CD4 TEM	CD4+, FOXP1-, INPP4B+, CCR7-, LEF1-
CD4 TSCM	CD4+, FOXP1+, LEF1+
CD8 Naive	CD8A+, CCR7+
CD8 TEM	CD8A+, CCR7-
HSPC	CD34+
MAIT	ME1+, SLC4A10+
NK	NCAM1+, CD38+
Plasma	CD38+, CD19+, CD79A+
Treg	CCR4+, IL32+, IL2RA+, FOXP3+
cDC	CD1C+, CD33+
gdT	CST7+
pDC	ZFAT+, CD4+

Table S4: A summary of marker genes and proteins used in the cell-type annotation of the RNA+Protein data of human PBMCs.

Cell type	Marker gene	Marker protein
B Memory	CD19,CD79A+,BANK1+	
B Naive	CD19,CD79A+,BANK1+,CD44+,SYN3+	
B CD3+	CD19,CD79A+,CD44+	CD3+
Basophil	RAB31+	
CD14 Mono	S100A8+, CSF3R+, VCAN+	CD14+
CD14 Mono(igG+)	S100A8+, CSF3R+, VCAN+	CD14+, igG1+, igG2a+, igG2b+
CD14 Mono(RA+)	S100A8+, CSF3R+, VCAN+	CD14+, CD45RA+
CD16 Mono	FCGR3A+, FCGR1G+	CD16+
CD4 Naive	CD4+, LEF1+	
CD4 TCM	CD4+, CCR7+, IL2RA-, FOXP1+	
CD4 TEM(RA+)	FOXP1-, INPP4B+, CCR7-, LEF1-	CD4+, CD45RA+
CD4 TEM(RO+)	FOXP1-, INPP4B+, CCR7-, LEF1-	CD4+, CD45RO+
CD4 TSCM	CD4+ , FOXP1+, LEF1+	
CD8 Naive	CCR7+	CD8a+
CD8 TEM	CCR7-	CD8a+
Inte Mono	S100A8+, CSF3R+, VCAN+	CD14+, CD16+
MAIT	ME1+, SLC4A10+	
NK	NCAM1+, CD38+	
Plasma	CD38+, CD19+, CD79A+	
Treg	CCR4+, IL32+, IL2RA+, FOXP3+	
cDC	CD1C+, CD33+	
gdT	CST7+, S100B+	
pDC	ZFAT+, CD4+	

Table S5: A summary of marker genes and proteins used in the cell-type annotation of the RNA+Protein data of human bone marrow.

Cell type	Marker gene	Marker protein
CD4 Memory	IL7R+,CCR7+,CD27+	
CD4 Naive	SELL+,IL7R+,FHIT+	
CD8 Naive+	CD8B+,CD8A+,LEF1+	
CD56 bright NK	NCAM1+	
NK	GNLY+,NKG7+,GZMB+	
CD8 Meomory	CD127+	
CD8 Effector	KLRG1+,CD69+,CCL4+	
MAIT	SLC4A10+,KLRB1+	
gDT	TRDC+,GNLY+	
GMP	MPO+,RNASE2+	
cDC2	FCER1A+,CLEC10A+	
CD16 Mono	FCGR3A+,IFITM3+,LST1+	
CD14 Mono	CD14+,S100A8++,LTB+	
pDC	IL3RA+,CLEC4C+	
Red blood cell	HBB+,GYPA+,GATA1+,MYC+	
Prog_B	VPREB1+,IGLL1+	
LMPP	CD34+,SPINK2+	
Plasmablast	MZB1+,SSR4+,FKBP11+	
Memory B	CD27+,MS4A1+,CD37+,CD52+	
Naive B	FCER2+	

Table S6: Running time used by VIMCCA, Seurat v4, totalVI, CiteFuse and DEMOC on six data sets with 3,000, 6,000, 9,000, 12,000, 15,000 and 18000 cells, respectively (unit: Second).

Cells	VIMCCA	totalVI	Seurat4.0	CiteFuse	DEMOC
3000	49	95	17	1046	3903
6000	82	203	30	8318	6117
9000	107	194	46	27338	>2h
12000	135	304	69	64690	
15000	161	321	83	>24h	
18000	191	608	112		

Table S7: Peak memory usage of VIMCCA, Seurat v4, totalVI on six data sets with 3,000, 6,000, 9,000, 12,000, 15,000 and 18000 cells, respectively (unit: MB).

Cells	VIMCCA	totalVI	Seurat4.0
3000	6032	3729	2944
6000	6664	3789	4502
9000	7252	3856	4958
12000	7858	3920	7137
15000	8415	3983	8476
18000	8954	4047	9980

Supplementary Figures

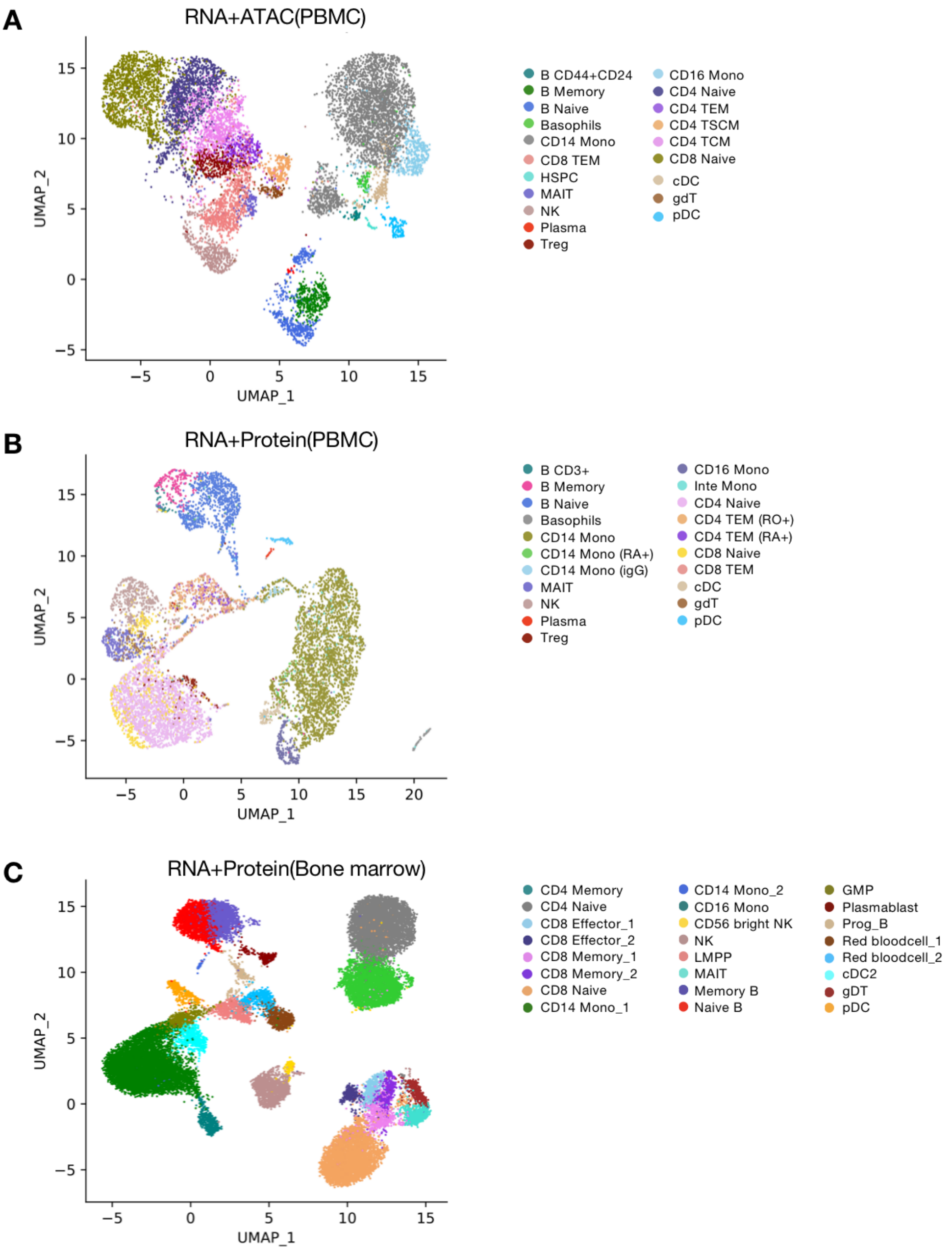

Figure S1: **UMAP visualization obtained by VIMCCA without dropout layers.** (A-C) UMAP visualization of joint-modality data (RNA+ATAC) integrated by VIMCCA without dropout layers. (B) UMAP visualization of joint-modality data (RNA+Protein) integrated by VIMCCA without dropout layers. (C) UMAP visualization of joint-modality data (bone marrow) integrated by VIMCCA without dropout layers.

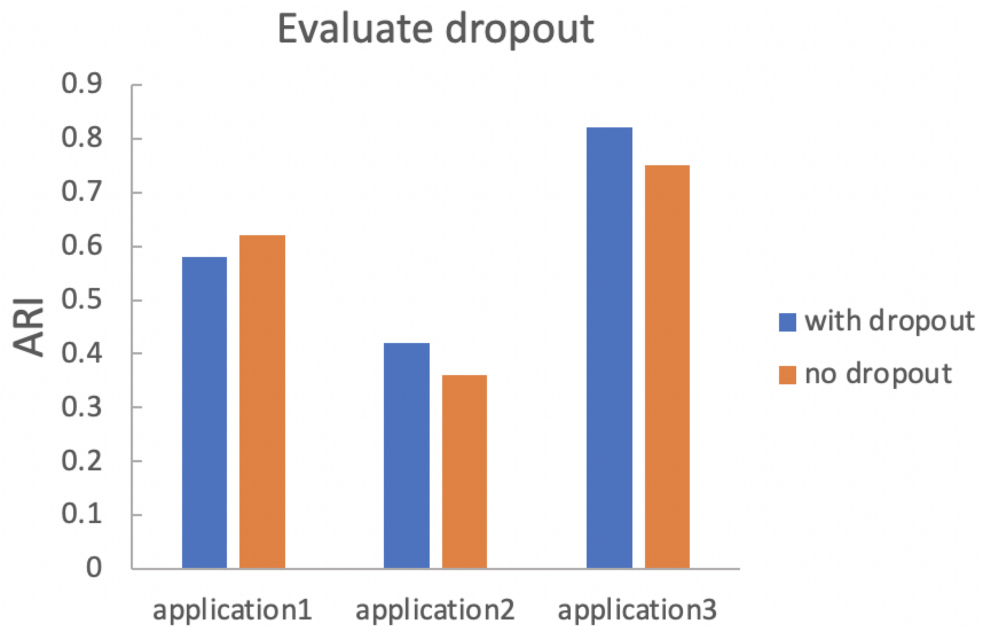

Figure S2: Performance comparison between VIMCCA with dropout and VIMCCA without dropout in terms of ARI.

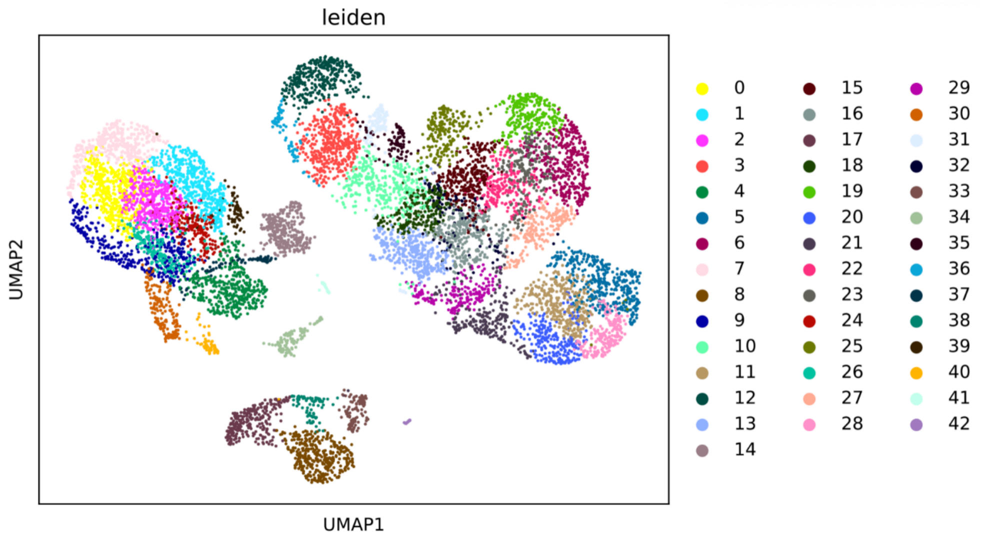

Figure S3: The 42 clusters obtained by VIMCCA on the 10X Multiome ATAC+RNA dataset of 10,412 paired human PBMCs. The Leiden algorithm was used for clustering.

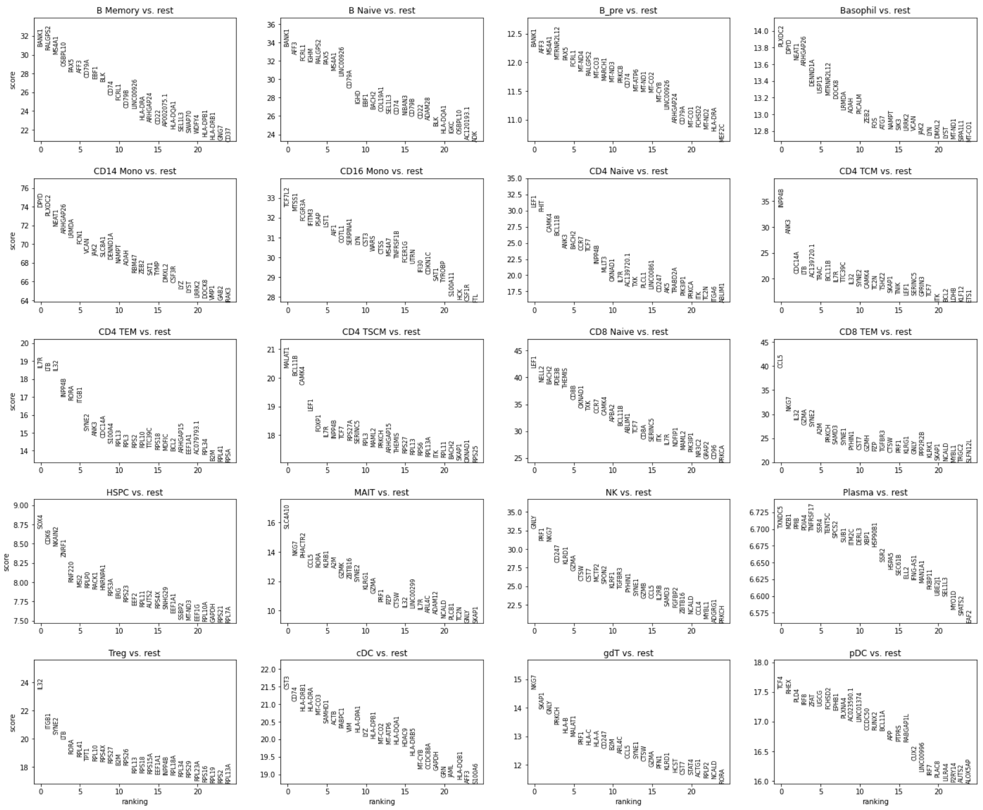

Figure S4: **Differentially expressed genes for each cluster**. We performed the Wilcoxon test to find differentially expressed genes in each cluster identified by VIMCCA for the ATAC+RNA dataset.

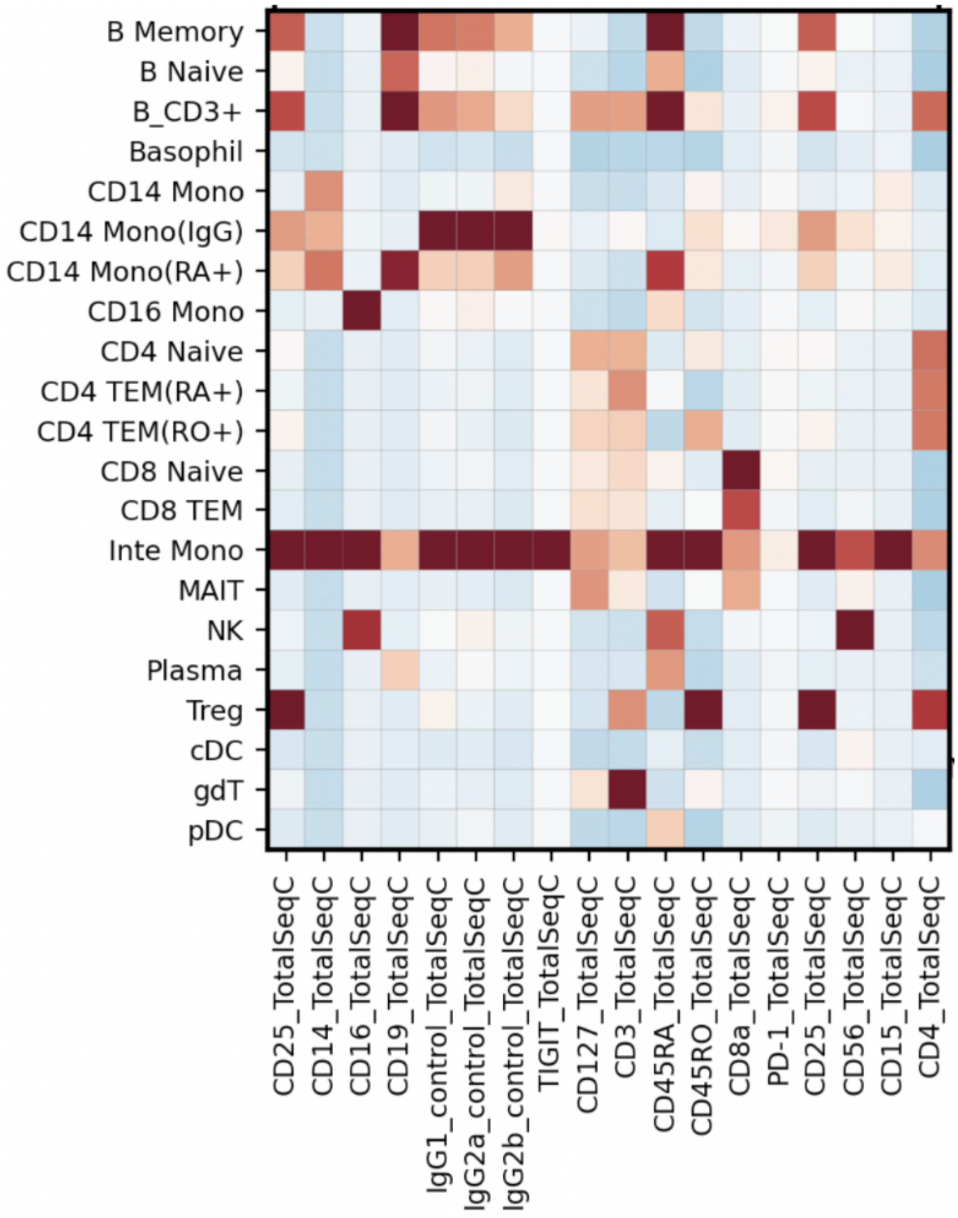

Figure S5: Heatmap of 17 protein expressions patterns in each cell type for the RNA+Protein data.

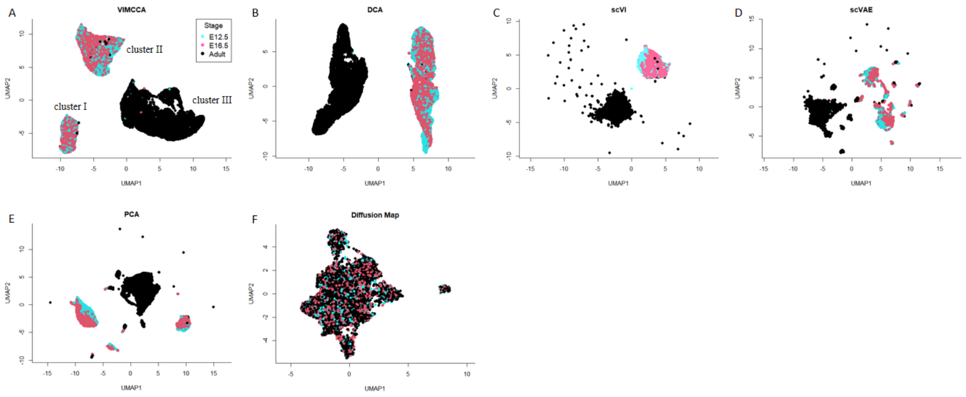

Figure S6: Visualization of cell representation obtained by eight algorithms. Cells are colored by the sampling stage. The blue, pink, and black cells indict that cells are sampled from E12.5, E16.5, and adult mice, respectively.

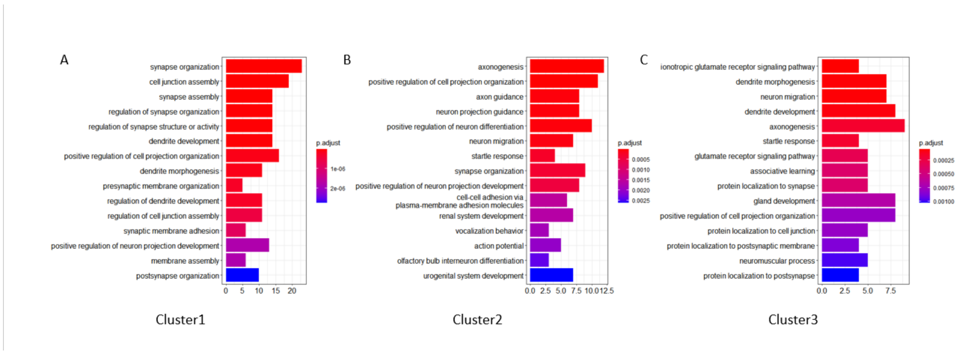

Figure S7: **Gene set enrichment analysis for differentially expressed genes in the aspect of biological process (BP)**. (A) Enrichment analysis for differential expression genes of the (VIMCCA) cluster at the lower left. (B) Enrichment analysis for differential expression genes of the (VIMCCA) cluster at the upper left. (C) Enrichment analysis for differential expression genes of the (VIMCCA) cluster at the lower right.

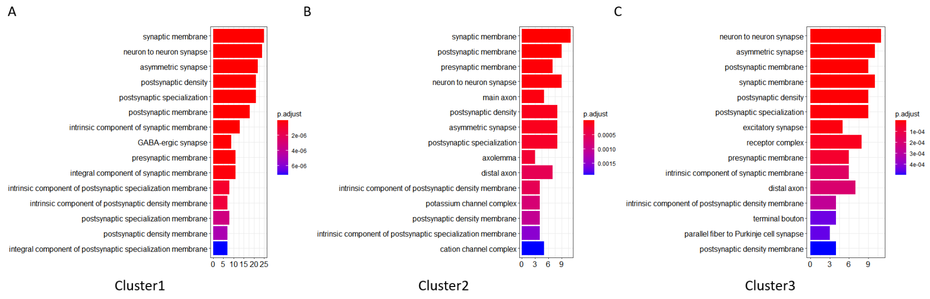

Figure S8: **Gene set enrichment analysis for differentially expressed genes in the aspect of cellular component (CC)**. (A) Enrichment analysis for differential expression genes of the (VIMCCA) cluster at the lower left. (B) Enrichment analysis for differential expression genes of the (VIMCCA) cluster at the upper left. (C) Enrichment analysis for differential expression genes of the (VIMCCA) cluster at the lower right.

References

[1] Hao Y, Hao S, Andersen-Nissen E, Mauck III WM, Zheng S, Butler A, et al. Integrated analysis of multimodal single-cell data. Cell. 2021.

[2] Gayoso A, Steier Z, Lopez R, Regier J, Nazor KL, Streets A, et al. Joint probabilistic modeling of single-cell multi-omic data with totalVI. Nature Methods. 2021;18(3):272-82.

[3] Lopez R, Regier J, Cole MB, Jordan MI, Yosef N. Deep generative modeling for single-cell transcriptomics. Nature methods. 2018;15(12):1053-8.

[4] Grønbech CH, Vording MF, Timshel PN, Sønderby CK, Pers TH, Winther O. scVAE: variational auto-encoders for single-cell gene expression data. Bioinformatics. 2020;36(16):4415-22.

[5] Gayoso A, Lopez R, Xing G, Boyeau P, Valiollah Pour Amiri V, Hong J, et al. A Python library for probabilistic analysis of single-cell omics data. Nature Biotechnology. 2022;40(2):163-6.

[6] Wu KE, Yost KE, Chang HY, Zou J. BABEL enables cross-modality translation between multiomic profiles at single-cell resolution. Proceedings of the National Academy of Sciences. 2021;118(15).

[7] Gong B, Zhou Y, Purdom E. Cobolt: integrative analysis of multimodal single-cell sequencing data. Genome biology. 2021;22(1):1-21.

[8] Argelaguet R, Arnol D, Bredikhin D, Deloro Y, Velten B, Marioni JC, et al. MOFA+: a statistical framework for comprehensive integration of multi-modal single-cell data. Genome biology. 2020;21:1-17.

[9] Zuo C, Chen L. Deep-joint-learning analysis model of single cell transcriptome and open chromatin accessibility data. Briefings in Bioinformatics. 2021;22(4):bbaa287.

[10] Ashuach T, Reidenbach DA, Gayoso A, Yosef N. PeakVI: A deep generative model for single-cell chromatin accessibility analysis. Cell reports methods. 2022;2(3):100182.

[11] Kim HJ, Lin Y, Geddes TA, Yang JYH, Yang P. CiteFuse enables multimodal analysis of CITE-seq data. Bioinformatics. 2020;36(14):4137-43.
